# Isolation and Diversity Analysis of Resistance Gene Homologues from Switchgrass

**DOI:** 10.1534/g3.112.005447

**Published:** 2013-06-01

**Authors:** Qihui Zhu, Jeffrey L. Bennetzen, Shavannor M. Smith

**Affiliations:** * Department of Genetics, The University of Georgia, Athens, Georgia 30602; † Department of Plant Pathology, The University of Georgia, Athens, Georgia 30602

**Keywords:** resistance gene homologue (RGH), genetic diversity, population structure, switchgrass, bioenergy crop, NBS-LRR

## Abstract

Resistance gene homologs (RGHs) were isolated from the switchgrass variety Alamo by a combination of polymerase chain reaction and expressed sequence tag (EST) database mining. Fifty-eight RGHs were isolated by polymerase chain reaction and 295 RGHs were identified in 424,545 switchgrass ESTs. Four nucleotide binding site−leucine-rich repeat RGHs were selected to investigate RGH haplotypic diversity in seven switchgrass varieties chosen for their representation of a broad range of the switchgrass germplasm. Lowland and upland ecotypes were found to be less similar, even from nearby populations, than were more distant populations with similar growth environments. Most (83.5%) of the variability in these four RGHs was found to be attributable to the within-population component. The difference in nucleotide diversity between and within populations was observed to be small, whereas this diversity is maintained to similar degrees at both population and ecotype levels. The results also revealed that the analyzed RGHs were under positive selection in the studied switchgrass accessions. Intragenic recombination was detected in switchgrass RGHs, thereby demonstrating an active genetic process that has the potential to generate new resistance genes with new specificities that might act against newly-arising pathogen races.

Switchgrass (*Panicum virgatum* L.) is a perennial grass that is native to North and Central American tallgrass prairies. Switchgrass will grow under a wide range of climatic conditions and has demonstrated high productivity in fields with marginal-to-poor soil quality while requiring low water, fertilization, and herbicide inputs (McLaughlin *et al.* 1999). Thus, switchgrass has attracted increasing attention as a potential source of biomass feedstock for renewable energy production (Sanderson 2000).

Although different growth habits and morphology allow most switchgrass accessions to be classified as either upland or lowland ecotypes, intermediate types are also observed (Hultquist *et al.* 1996, 1997). Lowland ecotypes are commonly tetraploid and found in wetter environments, whereas upland ecotypes are commonly hexaploid or octoploid and found in drier environments (Brunken and Estes 1975; Hultquist *et al.* 1996).

Recently, several molecular studies have been conducted to analyze switchgrass diversity and relatedness using DNA markers (Gunter *et al.* 1996; Huang *et al.* 2003; Casler 2005; Missaoui *et al.* 2006; Narasimhamoorthy *et al.* 2008; Cortese *et al.* 2010). Random amplified polymorphic DNA and chloroplast DNA restriction fragment length polymorphism markers revealed a high differentiation between upland and lowland ecotypes, but variation was not associated with ploidy level (Gunter *et al.* 1996; Casler 2005; Missaoui *et al.* 2006; Cortese *et al.* 2010). Similar conclusions were reported for expressed sequence tag (EST)-simple sequence repeat analysis of 31 switchgrass populations, where the upland and lowland accessions clustered into separate groups and also demonstrated clustering of populations based on their geographic origins (Narasimhamoorthy *et al.* 2008). In addition, combined data from molecular and morphological markers determined that the majority of the molecular variation (64%) in 12 New Jersey switchgrass populations existed within populations, whereas 36% was between populations (Cortese *et al.* 2010). These studies provide valuable information for researchers working with switchgrass but do not broadly investigate either the molecular nature of the genetic diversity observed or the pertinence of this diversity to particular genes that affect the fitness of switchgrass varieties.

One vital trait that affects all plants grown in agricultural or natural environments is their ability to withstand disease. Plants use several different types of disease-resistance genes to detect the presence of pathogens and induce defense responses. The most abundant resistance genes are those that encode proteins with nucleotide binding site (NBS) and leucine-rich repeat (LRR) domains (Bent 1996; Hulbert *et al.* 2001). Approximately three fourths of the plant disease resistance genes that have been cloned to date are from this class. The NBS domain contains several conserved motifs that are responsible for nucleotide binding and initiating a signal transduction cascade to activate plant defenses (Tameling *et al.* 2002). The LRR region is typically involved in protein−protein interactions and pathogen recognition specificity (Kobe and Deisenhofer 1995; Leister and Katagiri 2000; Dangl and Jones 2001). In addition, both domains are involved in intramolecular interactions to optimize detection of the pathogen and may be central to the control of *R*-gene activation (Bendahmane *et al.* 2002; Moffett *et al.* 2002; Hwang and Williamson 2003; Ravensdale *et al.* 2012).

Whole-genome sequencing of model plant species has enabled genome-level investigations of the resistance gene homologs (RGHs) in monocot and dicot species, such as Arabidopsis (Meyers *et al.* 2003; Tan *et al.* 2007), rice (Monosi *et al.* 2004; Zhou *et al.* 2004; Yang *et al.* 2008a), poplar (Kohler *et al.* 2008; Yang *et al.* 2008b), grape (Yang *et al.* 2008a), Medicago (Ameline-Torregrosa *et al.* 2008), sunflower (Radwan *et al.* 2008), and sugarcane (Glynn *et al.* 2008). These studies have demonstrated that RGHs are abundant in many plant genomes. For example, approximately 174 RGHs were identified in Arabidopsis, 519 in rice, 416 in poplar, and 535 in grape. Molecular genetic analysis has also shown that RGHs tend to occur in clusters in plant genomes (Kanazin *et al.* 1996; Collins *et al.* 1998; Young 2000; Donald *et al.* 2002; Ashfield *et al.* 2003; Calenge *et al.* 2005; Welter *et al.* 2007). Although the availability of RGH sequences from various plant species has enabled genome-level investigation of RGHs, there are only a few examples of studies analyzing resistance genes at the population level (Ramakrishna *et al.* 2002; Bakker *et al.* 2006; Ding *et al.* 2007; Rose *et al.* 2007; Caicedo 2008; Rouse *et al.* 2011).

Comparative analysis of RGHs in different plant species has provided evidence that R-genes are subject to positive selection, particularly in the LRR region. The LRR encodes solvent exposed residues that are predicted to interact either directly or indirectly with the corresponding *Avr* protein in the pathogen (Kobe and Deisenhofer 1995; Dangl and Jones 2001). Sequence comparisons among several groups of Arabidopsis NBS-LRR gene family members demonstrated that selection has acted to diversify the LRR domain (Mondragón-Palomino *et al.* 2002). Moreover, analysis of tomato and Arabidopsis RGHs revealed remarkably rapid evolution of RGHs during the radiation of plant families (Pan *et al.* 2000). Comparative studies of R-genes from tomato, lettuce, rice, flax, and Arabidopsis have demonstrated that solvent-exposed positions of the LRRs are hypervariable (Mondragón-Palomino *et al.* 2002) and suggest that selective forces imposed by the pathogen incite allelic diversity (Hulbert *et al.* 2001). Therefore, the selective advantage of carrying an R-gene and the pressure imposed on the R-gene to diversify depends on the frequency of the corresponding *Avr* gene in the pathogen population. Bergelson *et al.* (2001) have shown that balancing selection for rare RGH alleles competes with selective sweeps caused by the arms race with dominant pathogen races to create a very dynamic RGH repertoire (Bergelson *et al.* 2001). Hence, investigation of RGH distribution and variation patterns has proven to be a powerful tool to estimate R-gene abundance and selection pressure.

There is currently a project underway to sequence the switchgrass genome (http://www.phytozome.net) targeted on AP13, a tetraploid lowland cultivar. In theory, mining the shotgun sequencing data for RGHs could be useful, but there is a significant problem with the collapse of homologous domains into a single assembly with variability that cannot be attributed to any specific haplotype (Bennetzen *et al.* 2012). This is particularly problematic with repeated gene families like those routinely found for RGHs. A shotgun analysis can reduce as many as dozens of copies of highly similar genes to a single chimeric assembly. Hence, to study specific haplotypes, it was decided that a polymerase chain reaction (PCR)-based approach was most appropriate, where single molecules would represent pertinent regions of each haplotype. However, because EST analysis can yield chimeric assemblies and template switching can also occur during PCR, we decided to concentrate on a combined approach that featured both fosmid-based genomic cloning and mining of EST resources. These two independent strategies should complement each other, to provide the broadest discovery potential and to allow identification of any technical artifacts that were represented in only one discovery pipeline.

Although a candidate sustainable bioenergy crop, switchgrass has not yet been grown in monoculture over thousands of acres, so it is not known which pests or pathogens will ultimately most affect this crop. Pathogens could become the major limiting factor to seedling establishment, biomass quality, and/or yield in switchgrass, which has been the case for other crops, especially grasses. Analysis of R-genes in the switchgrass germplasm will help identify resistance gene haplotypes that will be vital for the production of disease and pest resistant varieties. The specific objectives of this study were to develop tools to isolate and sequence resistance gene homologs from switchgrass, to analyze the haplotype diversity of RGH loci in the switchgrass germplasm collection and to postulate how these RGHs evolve. This study describes the diversity and population structure of four RGHs from seven representative switchgrass populations. The findings provide insights into switchgrass RGH abundance and variability, thus generating a data resource for future use of this class of genes for improved switchgrass cultivar performance.

## Materials and Methods

### Plant material

Sixty-two switchgrass individuals from seven populations were used for this study. Five of the populations (collected from Kansas [KS], New Mexico [NM], North Carolina [NC], South Dakota [SD] and Texas [TX]) were obtained from GRIN (Germplasm Resources Information Network, USDA), and two Florida populations (FL1 and FL2) were obtained from the Brooksville Plant Materials Center in Brooksville, Florida (Table 1). The populations were first selected based on their ecotype, including three upland ecotypes, three lowland ecotypes, and one intermediate type (Table 1). The seven populations were then further separated into four groups, with one to two populations in each group, based on their origin north or south of 40°N latitude. Upland and lowland ecotypes originating North of 40°N latitude were designated Northern-upland and Northern-lowland, respectively (Casler *et al.* 2004), whereas upland and lowland ecotypes originating South of 40°N latitude were designated Southern-upland and Southern-lowland, respectively (Table 1). Genomic DNA was isolated from fresh leaf tissue collected from the 62 switchgrass individuals using the CTAB method described by Murray and Thompson 1980.

**Table 1:** Switchgrass plant material used in this switchgrass diversity study

Population	Accession	Ecotype	Ecotype Group	Origin	Number of Individuals
FL1	HSP	Lowland	SL	Florida, USAA	9
FL2	Pasco Co	Upland	SU	Florida, USA	10
KS	PI 421521 (Kanlow)	Lowland	NL	Kansas, USA	10
NC	PI 414067	Intermediate	−	North Carolina, USA	7
NM	PI 414066	Upland	SU	New Mexico, USA	8
SD	PI 642191 (Summer)	Upland	NU	South Dakota, USA	9
TX	PI 422006 (Alamo)	Lowland	SL	Texas, USA	9

NU, Northern upland; SU, Southern upland; NL, Northern lowland; SL, Southern lowland.

### PCR amplification using degenerate primers and NBS fragment isolation

Nine combinations of degenerate primers were designed (Supporting Information, Table S1) from four conserved motifs (P-loop, Kinase, MHD, and GLPL) within the NBS domain of resistance proteins (Meyers *et al.* 2003). These motifs have been used in the design of PCR-based cloning and mapping strategies to characterize R-genes from dicot and monocot species. Three forward primers targeted the P-loop (GGVGKTT) or kinase motif and eight reverse primers targeted the GLPL (GLPLAL) motif or MHD motif within the NBS domain.

The NBS domain of RGHs was amplified from the Alamo AP13 switchgrass accession (PI 422006) with degenerate primers. PCR was performed in 50-μL reactions with 10 mM Tris-HCl, pH 8.8; 50 mM KCl; 1.5−2.5 mM MgCl_2_; 0.8 mM total deoxyribonucleotide triphosphates; 4U of High Fidelity Taq DNA polymerase; 25 pmol of a forward and reverse degenerate primer; and 50 ng of genomic DNA. An annealing temperature gradient was initially used that ranged from 50° to 56° to optimize the annealing temperature for different primer pair combinations. PCRs were performed on a PTC-gradient cycler (MJ Research). The resulting PCR products were isolated from a 1.5% agarose gel, purified with an Invitrogen Quick gel extraction kit (Carlsbad, CA), and cloned into the Invitrogen pCR2.1-TOPO cloning vector using the methods described by the manufacturer. Six to eight clones were sequenced per gel purification product using the Big-Dye Terminator v3.1 cycle sequencing kit (Applied Biosystems), following the manufacturer’s protocol.

Sequences were aligned using ClustalW2 (Larkin *et al.* 2007). A BLASTN search was performed against the National Center for Biotechnology Information (NCBI) nonredundant database to verify putative homologies to known RGHs. A phylogenetic tree was constructed with all putative RGH sequences by MEGA 5 (Tamura *et al.* 2011) using the Neighbor-Joining (NJ) method. Based on the phylogenic relationship of the RGHs, an NBS-LRR coding sequence was selected from each phyletic group corresponding to switchgrass RGH (SwR) families. Sequences from the SwR family were used as a query to run BLASTN against a switchgrass EST database and to design specific primers to screen a switchgrass fosmid library.

### Mining switchgrass EST databases for NBS-LRR encoding resistance gene homologs

A switchgrass EST database containing 424,545 ESTs was provided by the Department of Energy, Joint Genome Institute. NBS-LRR coding sequences from each SwR family described in the previous section was used as a query (BLASTN and TBLASTX) to search the switchgrass EST database for new NBS-LRR RGHs not found in the SwR families. Additionally, the switchgrass EST database was mined to search for NBS-LRR encoding resistance genes by Hidden Markov Model (HMM, http://hmmer.janelia.org/) and GeneWisedb (Birney and Durbin 2000) approaches. The HMM approach translated the 424,545 switchgrass EST reads into six reading frames, using the TRANSEQ program of EMBOSS package (Rice *et al.* 2000). HMMER and GeneWisedb searches were performed by NB-ARC Pfam HMM PF00931 to identify switchgrass EST sequences encoding an NBS domain. To confirm the identification of sequences containing the NBS domain, sequences of the predicted NBS-containing proteins were used to run BLASTP against the NCBI protein nonredundant database (Altschul *et al.* 1997). The amino acid sequence of the confirmed NBS ESTs was aligned using ClustalW2 (Larkin *et al.* 2007). Phylogenetic tree construction was performed with MEGA version 5 (Tamura *et al.* 2011) to identify new NBS-LRR clusters. Based on phylogenetic relationships, ESTs were selected from phyletic groups that were homologous to known NBS genes in other species. Sequences from the EST families were used to design specific primers to screen a switchgrass fosmid library by PCR.

### Fosmid genomic library screening

A switchgrass genomic fosmid library was screened with a PCR-based approach. The switchgrass fosmid library was constructed using the Alamo AP13 (PI 422006) accession (J. Hawkins, R. Percifield, and J. Bennetzen, unpublished data). The library coverage is approximately five times the switchgrass genome size (~1300 Mb) and is arranged into 34 superpools. Each superpool contains 48 pools. The switchgrass fosmid library was screened in four steps. (1) DNA from the 34 superpools was amplified with specific primers designed from the SwR and EST families that were homologous to known NBS genes in other plant species (Table S1). (2) Once a superpool was identified as positive in the first step, the 48 pools contained in the positive superpool were then amplified with these same primers. (3) A total of 96 randomly selected clones from each positive pool were amplified with the degenerate primers. In most cases, 96 clones were sufficient to identity at least one positive clone. If all of the 96 randomly selected clones showed negative amplification, an additional 96 clones were selected to further search for positive clones. (4) PCR amplification products from any positive clones among the 96 individual clones were sequenced, and BLASTX was run against the NCBI nonredundant database to verify that RGHs had been amplified. The PCR fragments with homology to NBS-LRR R-genes thereby identified the switchgrass fosmids that had target R-genes. Fosmids with full-length RGHs, identified by amplifying with primers that covered the full-length genes, were then sequenced from both ends using the Big-Dye Terminator v3.1 cycle sequencing kit (Applied Biosystems) following the manufacturer’s protocol.

Fosmid structure was annotated with the assistance of two *ab initio* gene finding programs, FgeneSH and GeneMark.hmm (Lukashin and Borodovsky 1998). Each gene prediction algorithm has its strengths and weaknesses; therefore, combining results from two gene finding programs can improve the quality of a prediction. Based on a previous study in maize, FgeneSH and GeneMark.hmm together yielded the most accurate gene predictions (Yao *et al.* 2005). For this work, a HMMER search performed with a NB-ARC Pfam HMM PF00931 was used to identify sequences encoding an NBS domain. If a domain was identified by HMMER search, but a full-length gene was not predicted by FgeneSH/GeneMark.hmm in the same region, then the resistance gene was designated a partial NBS-RGH.

### Cloning and sequencing from population samples

The PCR-based approach and switchgrass EST database search methods were used to identify RGHs for further analysis. The SwR and EST RGH families identified with the two aforementioned methods, respectively, were selected for diversity analysis in the seven switchgrass populations. Sequences from each SwR and EST family with an intact coding region and high similarity to known disease resistance genes in other grasses were aligned and used to design primers (Table S1). To exclude the possibility of amplifying paralogous sequences among individuals, we designed the forward primers in the conserved NBS and reverse primers in the diversified LRR regions. In addition, we ran BLAST searches against the NCBI database using the targeted PCR products as queries to further make sure there were no paralogous copies of the sequences in any species. The primers for each RGH also yielded a maximum of four different sequences when used on DNA from a single plant, which is the result expected for a highly heterozygous tetraploid if the gene amplified was single copy per haploid genome. PCR products from the seven switchgrass populations ranged from 896 to 1023 bp. PCR products were gel excised, purified, and cloned as described previously. Six to eight independent plasmids were selected randomly for each population and sequenced using the Big-Dye Terminator v3.1 cycle sequencing kit (Applied Biosystems) following the manufacturer’s protocol. All sequencing was performed at the UGA sequencing facility and sequences were submitted to GenBank (Accession numbers: JN231541-JN232038; Table S3).

### Sequence diversity analyses

Sequence diversity in more than 300 RGHs from switchgrass was analyzed for four representative loci (SwPc, SwPI, SwMLA, and SwRIII). Sequences were aligned using a combination of methods implemented in ClustalW2 (Larkin *et al.* 2007) and BioEdit version 7.0.9 (Hall 1999), with further manual corrections of alignments. Sequences were analyzed using BLASTN to verify amplification of the correct gene and putative homologies of the sequences with resistance genes characterized in other plant species. Phylogenetic tree construction was performed with MEGA version 5 (Tamura *et al.* 2011) using the NJ method (Saitou and Nei 1987), with distances represented as the number of nucleotide differences. Confidence in the phylogeny was assessed with 1000 bootstrap replicates.

The number of segregating sites (*S*) and haplotypes (*h*) was calculated for each locus and population. Nucleotide diversity was estimated by calculating the average pairwise difference between sequences, *π* (Nei 1987), and the number of segregating sites in a sample, *θ_w_*. This parameter has an estimate of 4*N_e_*μ, where *N_e_* is the effective population size and *μ* is the mutation rate per nucleotide (Watterson 1975). Estimates of nucleotide diversity were based on total sequences and silent sites separately using DnaSP version 5.10.01 (Librado and Rozas 2009). The recombination parameter per gene and between adjacent sites was calculated based on the average number of nucleotide differences between pairs of sequences (Hudson *et al.* 1987). To measure the proportion of genetic variation between sites, *F*-statistics in analysis of molecular variance (AMOVA) were used. This analysis was performed using GenAlEx Ver 6.2 (Peakall and Smouse 2006) and tested the significance of all estimates based on 999 random permutations. A principal coordinate analysis (PCoA) graph was constructed for the four representative loci within the three switchgrass ecotypes and seven selected populations from various geographic origins (KS: Kansas; TX: Texas; FL1: Florida; SD: South Dakota; FL2: Florida; NM: New Mexico; NC: North Carolina).

To test for deviations from the neutral equilibrium model of evolution, Tajima’s *D* and Likelihood ratio (LR) analyses were performed using DnaSP and PAML (Yang 1997; Yang *et al.* 2000), respectively. Tajima’s *D* is based on the discrepancy between the mean pairwise differences (π) and Watterson’s estimator (*θ_w_*) (Tajima 1989). This parameter was calculated for each locus at all sites and at silent sites separately. LR analysis of positive selection, based on the maximum likelihood method and codon substitution models, was applied with the Codeml program Phylogenetic Analysis using Maximum Likelihood (PAML) (Yang 1997; Yang *et al.* 2000). The LR analysis compared neutral selection (M1) and positive selection (M2). M1 allows two ω site classes to be estimated from the data, ω_0_ < 1 or ω_1_ = 1. The ω parameter indicates the underlying nonsynonymous/synonymous rate ratio. M2 allows an additional ω site class value to be estimated from the data, ω_2_ > 1. When the LR analysis suggested that positive selection (>1) had occurred at any of the four representative loci, selected sites were further analyzed under the M2 model with the Bayesian approach implemented in PAML.

## Results

### Switchgrass RGHs identified by targeting conserved NBS sequence motifs and mining a switchgrass EST database

PCR amplification conditions were optimized for switchgrass using Alamo AP13 genomic DNA as template with different reaction mixtures and annealing temperatures for the nine degenerate primer pairs designed from four (P-loop, Kinase, MHD, and GLPL) conserved motifs within the NBS domain of resistance proteins (Table S1). PCR products ranging from ~300 bp to ~1500 bp were isolated and cloned as putative RGHs. Primers were designed using intron-lacking domains of known resistance genes. Sequences containing a continuous open reading frame encoding the amino acid motifs conserved in resistance proteins were designated RGHs. A total of 152 amplicons were cloned and sequenced, of which 88 amplicons were observed to be homologous to NBS sequences of NBS-LRR encoding genes previously isolated from other plants. The other 64 sequenced clones did not have a significant RGH BLAST homology.

**Figure 1:**
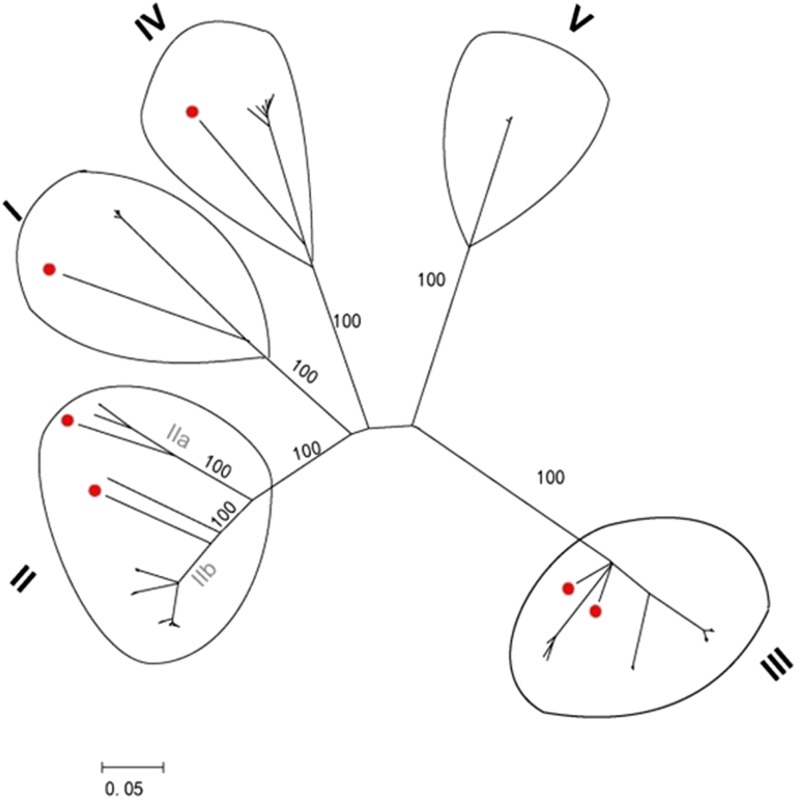
Unrooted NJ phylogenetic tree of switchgrass RGHs. Roman numerals at each cluster indicate putative switchgrass RGH classes (I, SwRI; II, SwRII; III, SwRIII; and IV, SwRIV), except class V, which has not been confirmed as an RGH family. Roman numerals with a letter indicate different RGH classes within a class. Red dots indicate R-genes from other species such as rice, wheat, and sugarcane. Numbers at nodes indicate the level of branch support (%) with 1000 bootstrap replicates.

Forty-six of the predicted 88 NBS-LRR encoding genes had uninterrupted open reading frames from the P-loop to the GLPLAL motif, whereas the remaining 42 amplicons harbored stop codons or frame shift mutations and were therefore designated as likely pseudogenes. Clones were defined as belonging to a cluster (that is, a closely related family of genes) when aligned sequences demonstrated at least 90% nucleotide identity. Bootstrap support for all of the clades was high (100%), indicating that these relationships are well-supported (Figure 1). As a result, five candidate RGH subfamilies were identified and designated SwRI, SwRII, SwRIII, SwRIV, and SwRV. The SwR designation indicates that the RGHs (R) were isolated from switchgrass (Sw), whereas the roman numerals correspond to the different RGH subfamilies. Four (I, II, III, and IV) of the five subfamilies clustered with R-genes from other species, such as rice, wheat, sugarcane, and barley, and were therefore designated putative switchgrass RGHs. These four SwR subfamilies were used to identify additional NBS-LRR coding genes from the switchgrass EST database search and the fosmid library screen. Class V did not cluster with any previously known R-genes and thus was not used for further analysis, although it may be targeted in future studies as a possible source of highly novel RGHs.

Sequences obtained from the HMMER and GeneWisedb searches were combined to identify 295 switchgrass NBS RGH sequences from 424,545 switchgrass ESTs. Among the switchgrass NBS RGHs, 203 were predicted as different contigs with ESTpass analysis (Lee *et al.* 2007). The phylogeny showed that the 295 NBS RGHs clustered into 211 groups with at least 50% similarity at the amino acid level. Sixty-one of the 295 NBS RGHs were organized into the four SwR subfamilies with an *E*-value of 1E-10 or below in the TBLASTX search. The remaining NBS RGHs did not cluster with the SwR subfamilies. However, 29 of the remaining NBS RGHs demonstrated high homology to RGHs from barley (*Mla*), rice (*pi*), sorghum (*Pc*), and wheat (*Yr10*) based on BLASTX run against the NCBI protein database. The barley *Mla* R-gene clustered with five switchgrass ESTs whereas *pi*, *Pc*, and *Yr10* formed a clade with six, eight, and 10 ESTs, respectively. Therefore, these four switchgrass EST subfamilies were designated SwMLA, SwPI, SwPc, and SwYr10. The Sw designation indicates that the ESTs were selected from switchgrass (Sw) and is followed by the name of the homologous R-gene. These four EST subfamilies were used for the switchgrass fosmid library screen and for the RGH diversity analysis.

### Structure and clustering of cloned RGHs

Twelve switchgrass fosmids were selected from many hundreds of PCR-positive clones amplified with RGH-targeting primers. A total of six PCR-positive fosmids corresponding to the SwR subfamilies were selected for further analysis. Three PCR positive fosmids, each corresponding to a different SwR subfamily (SwRI-1, SwRIII-1, and SwRIV-1), were selected (Figure 1). RGH sequences in the SwRII subfamily formed two distinct clades. Therefore, three PCR positive fosmids, one corresponding to one clade (SwRIIa-1) and two corresponding to the second clade (SwRIIb-1 and SwRIIb-2), were selected. A total of six PCR-positive fosmids corresponding to the EST subfamilies were also selected for further analysis. The fosmid insert sizes were found to be 40 kb or less, and inserts exhibited a combined GC content of 45.6%. Fgenesh predicted that each fosmid carries three to eight genes with an average gene density of one gene per 7 kb (Figure S1 and Table S2).

A total of 41 genes and 31 transposons were predicted to be on the 12 sequenced fosmids (Figure S1 and Table S2). Nineteen of the candidate genes corresponded to NBS-RGHs. Tandem arrays of RGHs were observed on five (SwPc-1, SwRIIa-1, SwRIIb-1, SwPI-1, and SwMLA-2) fosmids. Twenty-six of the transposons were LTR retrotransposons and five were DNA transposons. The predicted gene numbers do not include predicted transposon-encoded genes. Fosmid SwRIII-1 carried only three putative genes (Figure S1 and Table S2), whereas fosmids SwRI-1, SwRIIb-2, SwPc-1, and SwMLA-2 each contained eight predicted genes. Overall gene and transposon contents on these fosmids are presented in Figure S1 and Table S2.

### R-gene nucleotide diversity within populations

Four of the SwR (SwRI, SwRII, SwRIII, and SwRIV) and EST (SwMLA, SwPI, SwPc, and SwYr10) target NBS RGHs, SwRIII-1, SwPc-1, SwPI-1, and SwMLA-1 were highly similar to at least one fully sequenced disease resistance gene in other grasses. These fully sequenced genes were selected for diversity analysis because PCR primers could be designed to generate an amplification product that would cover the entire protein-encoding component of the gene. SwPc-1 is most homologous to the *Pc* locus in sorghum, which determines dominant sensitivity to a host-selective toxin produced by the fungal pathogen *Periconia circinata* (Nagy and Bennetzen 2008). SwPI-1, SwMLA-1, and SwRIII-1 are highly homologous to the blast resistance gene *pib* from rice (Wang *et al.* 1999), the powdery mildew resistance gene *Mla* from barley (Wei *et al.* 1999), and the Arabidopsis *RPM1* disease resistance gene (Grant *et al.* 1995), respectively. Sequences for each of the four RGH loci were amplified from the genomic DNA of 62 individuals derived from the seven switchgrass accessions (FL1: Florida; FL2: Florida; KS: Kansas; NC: North Carolina; NM: New Mexico; SD: South Dakota; TX: Texas), chosen for their representation of a broad range of the switchgrass germplasm. The seven populations represent the two major ecotypes (upland and lowland) and the intermediate type (Table 1). Multiple switchgrass individuals represent genetically distinct plants derived from a single accession. Therefore, the seven accessions were classified as representative switchgrass populations (Table 1).

Ten individuals were analyzed from the Kansas and Upland-Florida populations, whereas nine individuals were analyzed from the Lowland-Florida, South Dakota, and Texas populations. Eight and seven individuals were analyzed from the New Mexico and North Carolina populations, respectively. The length of aligned sequence for each locus varied between 814 bp and 1024 bp and contains only coding sites. A total of 24 polymorphic indels and 1205 single-nucleotide polymorphisms (SNPs) were detected. The lowland, upland, and intermediate ecotypes exhibited 550, 640, and 412 SNPs, respectively. Although numerous indels and SNPs were detected, most alleles of the four RGHs appeared to be functionally competent. In some cases, frame shifts or SNPs yielding premature stop codons suggested nonfunctional alleles, at levels of 14.6%, 14.3%, 9.7%, and 6.9% for the respective SwPc-1, SwPI-1, SwMLA-1, and SwRIII-1 genes. This may be an underestimate of the percentage of accessions containing nonfunctional alleles because missense mutations can also generate inactive proteins.

The genetic diversity of SwPc-1, SwRIII-1, SwMLA-1, and SwPI-1 was analyzed in the 62 switchgrass individuals to determine the genetic diversity of these RGHs in the seven switchgrass populations. The average nucleotide diversity (π) for the seven switchgrass populations was 0.51%, 2.74%, 3.50%, and 7.2% at SwPc-1, SwRIII-1, SwMLA-1, and SwPI-1, respectively (Table 2). Of the seven populations, the nucleotide diversity of the upland SD population was the lowest, 0.19% at the SwPc locus. Conversely, the lowland TX population harbored the highest diversity, 8.8% at the SwPI locus. The average number of different haplotypes for the seven populations was 74, 77, 112, and 146 at SwPc-1, SwMLA-1, SwRIII-1, and SwPI-1.

**Table 2:** Haplotype diversity of RGHs within switchgrass populations

		Number of Sequences				Number of Haplotypes*^a^*				π (%) Within Population*^b^*			
Population	Ecotype	SwPc	SwRIII	SwMLA	SwPI	SwPc	SwRIII	SwMLA	SwPI	SwPc	SwRIII	SwMLA	SwPI
FL1	Lowland	16	22	14	16	14	13	12	22	0.82	1.49	3.98	6.22
FL2	Upland	25	37	28	36	17	27	26	16	0.62	3.38	4.17	8.26
KS	Lowland	8	15	10	13	5	7	6	9	0.36	3.36	2.96	6.43
NC	Intermediate	17	24	8	26	17	20	6	23	0.57	2.36	3.72	7.34
NM	Upland	26	32	12	26	18	25	9	30	0.48	1.99	3.78	5.62
SD	Upland	17	21	17	26	8	13	11	25	0.19	2.82	2.92	7.73
TX	Lowland	18	27	18	36	14	25	16	34	0.51	3.77	2.95	8.80
Overall*^c^*						74	112	77	146	0.51	2.74	3.50	7.20

RGH, resistance gene homologs; FL, Florida; KS, Kansas; NC, North Caroline; NM, New Mexico; SD, South Dakota; TX, Texas.

a Number of unique haplotypes.

b Nucleotide diversity, the average number of nucleotide differences per site between two sequences (Nei 1987).

c Overall represents the total number of unique haplotypes for all samples considered together or the average π values across populations

Haplotypic diversity was also analyzed for the RGHs relative to ecotype (Table 3). The average nucleotide diversity for the four RGHs in the upland, intermediate and lowland ecotypes was 3.44%, 3.51%, and 3.98%, respectively. Supporting the RGH genetic diversity data for the seven switchgrass populations, the SwPc locus demonstrated the lowest diversity for the upland (0.41%), intermediate (0.39%), and lowland (0.68%) ecotypes and the SwPI-1 locus harbored the highest diversity for the three ecotypes (6.57%, 7.96%, and 7.75%). The average number of different haplotypes for the four RGHs was 21, 54, and 74 for the intermediate, lowland, and upland ecotypes, respectively. Interestingly, the nucleotide diversity observed for SwPc, SwRIII, and SwPI was very similar in the lowland and upland ecotypes when estimated with the *θ*_w_ parameter, whereas the diversity detected for the three RGHs in the same two ecotypes were quite different when using the π estimator (Table 3). SwMLA in the upland ecotypes was the exception, showing a much greater *θ*_w_ value. Watterson’s estimator (*θ*_w_) is a method used for estimating population mutation rate (genetic diversity) but also takes into account the effective population size and the mutation rate per-generation in the population of interest, whereas the π estimator is simply the sum of the pairwise differences divided by the number of pairs. Estimates of nucleotide variation in the NBS and LRR domains were detected for each locus. The level of nucleotide variation in the NBS domain was lower than that observed in the LRR domain (Table S3) when we compared domains of similar length.

**Table 3:** Haplotype diversity of RGHs in upland and lowland switchgrass ecotypes

	No. of Haplotypes*^a^*			π (%) Within Ecotypes*^b^*			*θ*_w_*^c^*		
Gene	Lowland	Upland	Intermediate	Lowland	Upland	Intermediate	Lowland	Upland	Intermediate
SwPc	28	27	7	0.68	0.41	0.39	0.88	0.80	0.39
SwRIII	24	30	16	4.18	2.79	1.81	3.43	3.24	3.52
SwMLA	45	59	21	3.31	4.00	3.88	3.47	5.48	3.15
SwPI	28	34	7	7.75	6.57	7.96	8.47	8.90	9.17
Overall*^d^*	38	49	16	3.98	3.44	3.51	4.06	4.61	4.06

a Number of unique haplotypes.

b Nucleotide diversity, the average number of nucleotide differences per site between two sequences (Nei 1987).

c *θ*_w_, 4*N*_e_u for an autosomal gene of a diploid organism (*N*_e_ and *u* are the effective population size and the mutation rate per DNA sequence per generation, respectively) (Tajima 1989).

***^d^***Overall represents the total number of unique haplotypes for all samples considered together or the average *π* or *θ*_w_ values across genes.

### RGH nucleotide diversity between and within populations

AMOVA, PCoA, and the Mantel test were used to estimate the genetic divergence between the seven populations and three ecotypes for the four RGH loci based on geographic origin. At two hierarchical levels, AMOVA revealed that an average of 16.5% of the genetic variation was attributed to between population diversity and 83.5% was attributed to within population diversity (Table S4). A nested analysis approach confirmed these data with 16.5% and 83.5% average diversity observed between and within populations, respectively (Table S5).

**Figure 2:**
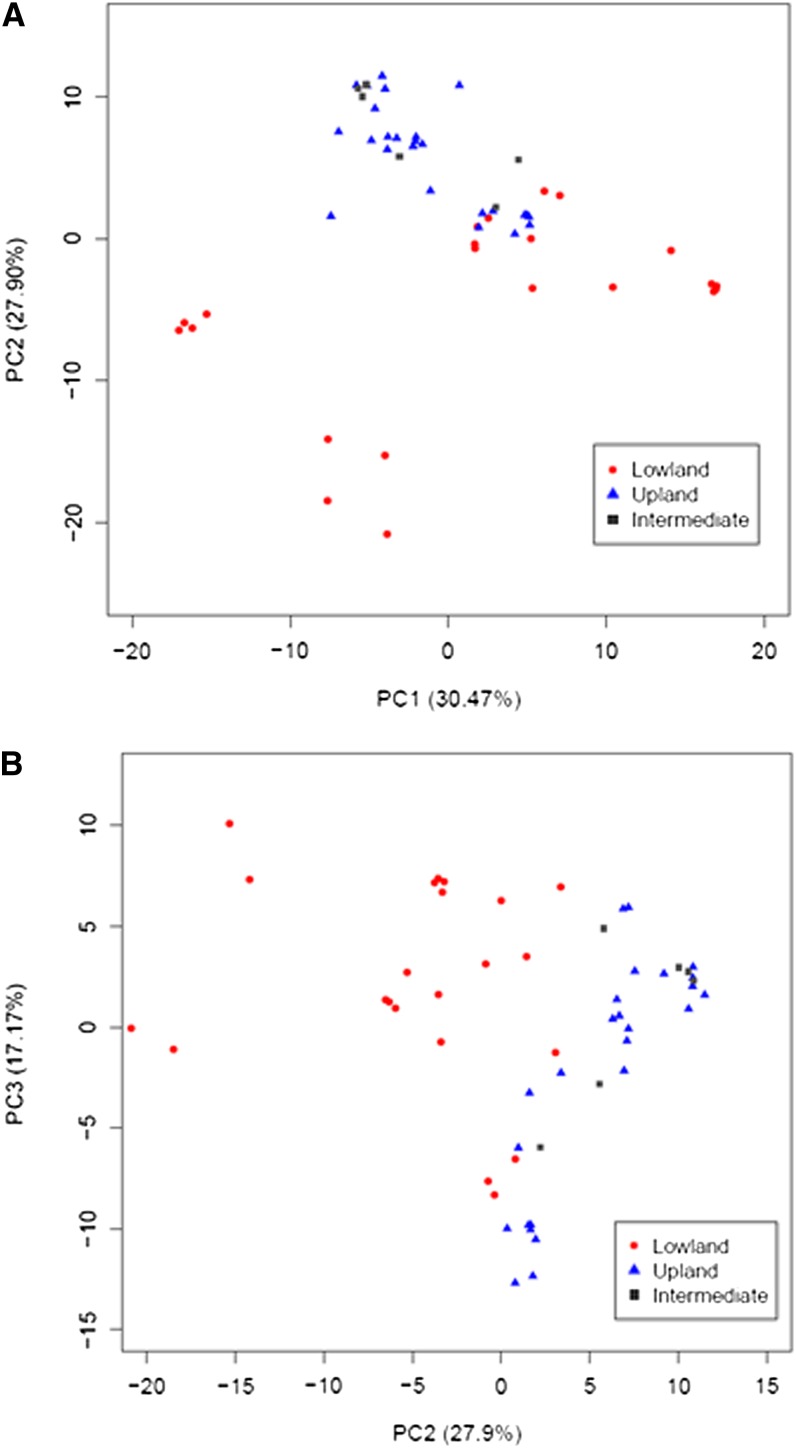
PCoA plots of RGHs for the lowland, upland, and intermediate switchgrass ecotypes. (A) Principal coordinates 1 *vs.* 2. The horizontal axis corresponds to the first component (PC1). The vertical axis corresponds to the second component (PC2). (B) Principal coordinates 2 *vs.* 3. The horizontal axis corresponds to the second component (PC2). The vertical axis corresponds to the third component (PC3). Negative and positive values on the vertical and horizontal axis are component scores and represent the transformed variable values corresponding to a particular data point. Percentage numbers on the vertical and horizontal axis in the parenthesis represent the proportion of variances explained by each component. Red dots represent the lowland ecotype, blue triangles represent the upland ecotype, and gray squares represent the intermediate ecotype.

**Figure 3:**
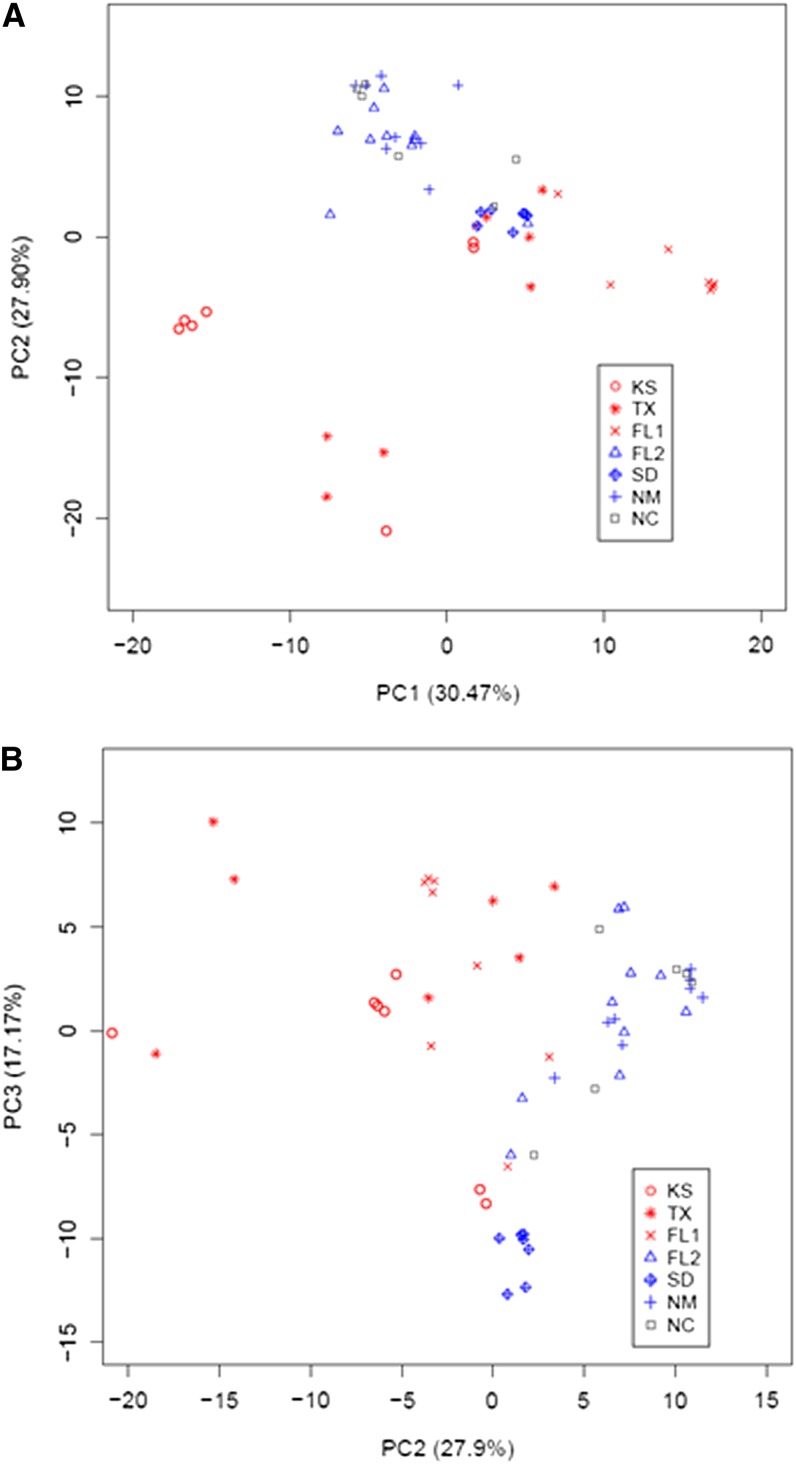
PCoA plot of RGHs for seven switchgrass representative populations. (A) Principal coordinates 1 *vs.* 2. The horizontal axis corresponds to the first component (PC1). The vertical axis corresponds to the second component (PC2). Seven signs represent seven populations sampled from various geographic origins (KS, Kansas; TX, Texas; FL1, Florida; SD, South Dakota; FL2, Florida; NM, New Mexico; NC, North Carolina). (B) Principal coordinates 2 *vs.* 3. The horizontal axis corresponds to the second component (PC2). The vertical axis corresponds to the third component (PC3). Negative and positive values on the vertical and horizontal axis are component scores and represent the transformed variable values corresponding to a particular data point. Percentage numbers on the vertical and horizontal axis in the parenthesis represent the proportion of variances explained by each component.

We performed PCoA based on the pairwise differences between individuals. The two-dimensional plot for PC1 (principal coordinate 1) and PC2 (principal coordinate 2) demonstrated differentiation along PC2 between lowland and upland ecotypes (Figure 2). Samples from each ecotype typically clustered together. Northern-upland and Southern-upland populations demonstrated an overlapping distribution, whereas samples of various haplotypes from the lowland ecotypes were loosely distributed along PC1. For example, a few of the haplotypes from the Northern-lowland Kanlow variety were the most distant from the Southern-lowland populations (Figure 3). In addition, the Mantel test, which evaluates the matrix of pairwise genetic distances against the matrix of pairwise geographic distances, did not detect a significant correlation between genetic distance and geographic distance (*r* = −0.0199, *P* = 0.094).

These results were supported by phylogenetic analysis of the RGHs characterized in the switchgrass populations (Figure S2). SwRIII-1 and SwMLA-1 RGHs did not cluster together on the same branch or on closely related branches. Only two distinct clades were observed for the SwPc-1 locus that demonstrated the lowest genetic diversity (Figure S2A and Figure S2B). In addition, all of the branch lengths were short with the exception of the out-group. In contrast, the more divergent RGHs, such as SwRIII-1 and SwPI-1, typically demonstrated several clades with long branches between allelic groups, but very short branches within a clade (Figure S2C and Figure S2D).

### Detection of positive selection and recombination

LR analysis was applied to test for positive selection based on the maximum likelihood method and codon substitution models with the Codeml program from PAML (Yang 1997, 2000). The LR tests identified two genes, SwMLA-1 and SwPI-1, that fit the selective model better than the null model (Table S6). When the LR test suggested positive selection had occurred, selected sites were further identified under the M2 model with the Bayesian approach implemented in PAML. Nine sites from SwPI-1 and four sites from SwMLA-1 were identified to be under significant positive selection. Most of the sites under selection at the SwPI-1 locus were located in the LRR region, while the four sites at SwMLA-1 were located outside of the LRR domain in the Loop region between the NBS and LRR domain (Table S7). Tajima’s *D* statistics was also used to detect natural selection. Tajima’s *D* distributions were skewed toward negative values for all four RGHs, indicating a relative excess of low frequency alleles compared with expectations under a stationary neutral model. When Tajima’s *D* tests were applied separately to the NBS and LRR regions, negative values were still observed with no selection detected with significant *P* values.

The frequency of recombination was examined for the four loci using the DNAsp recombination parameter (Hudson *et al.* 1987). The minimum number of recombination events between adjacent polymorphic sites for SwPc-1, SwRIII-1, SwMLA-1, and SwPI-1 were 7, 21, 23, and 62, respectively. The recombination rate between adjacent sites was greatest in SwPI-1 (0.0491), followed by SwMLA-1 (0.0285), SwPc-1 (0.0149), and SwRIII-1 (0). These high recombination frequencies indicated that recombination is generating a great deal of the genetic diversity in some RGHs.

## Discussion

### Structure and clustering of R-genes

Is this study, 88 RGHs were identified in the Alamo AP13 accession with the PCR-based approach and grouped into five clusters with RGHs only from cluster II having significant hits to the switchgrass EST database. The switchgrass ESTs were derived from several libraries developed from different tissues including callus, seedling, crown, stem, roots, and early-/late-flowering parts. A total of 295 RGHs were identified from 424,545 switchgrass ESTs with an EST database mining approach. This finding suggests that RGH expression levels are relatively low in switchgrass for RGH families I, III, and IV because these RGHs were found to be abundant in the switchgrass genome, yet no switchgrass ESTs were identified for these three RGH families. Previous work also suggests that only a few of the NBS-LRR−encoding RGHs in plants exhibit detected expression, and those at a fairly low level (Dilbirligi and Gill 2003; Monosi *et al.* 2004; Radwan *et al.* 2008). Of course, many more tissue types, developmental states and environmental conditions could be used as sources of RNA, and this would probably somewhat increase the number of RGHs with detected expression products, but the story would probably remain that this category of genes is several fold under-represented in expression data relative to average genes in every tissue sample examined. For example, in rice, 130 NBS-LRR encoding RGHs were identified from 28,000 full-length cDNA clones (Monosi *et al.* 2004), although 230 families of NBS-LRR encoding RGH sequences were annotated in the near-fully sequenced rice genome (Zhou *et al.* 2004).

Previous analysis of RGHs has demonstrated that plant disease resistance genes frequently occur in tightly linked clusters (reviewed in Michelmore and Meyers 1998). In maize, for instance, multiple *Rp* genes have been shown to mediate resistance to the fungal pathogen *Puccinia sorghi*. Fourteen genetically separate loci were mapped to this locus and designated the *Rp1* complex (Hulbert 1997). Complex disease resistance clusters have also been identified in Arabidopsis (Meyers *et al.* 2003), rice (Song *et al.* 1997), barley (Wei *et al.* 1999), and many other species. Several of the specificities within these genetically well-defined resistance loci have been targeted for molecular cloning and analysis. Similarly, we sequenced and characterized 12 switchgrass fosmids carrying full-length RGHs and found that five of the 12 fosmids carried more than one RGH, often in tandem arrays as seen in other species. The analysis was restricted to an insert size of 40 kb or less, so we expect that additional RGHs would be identified if a larger region of the haplotype were sequenced. Also, many transposable elements were identified in close proximity to RGHs in the switchgrass fosmids. Plant transposable elements can play a role in the evolution of resistance genes, primarily because their insertion nearby can create novelty in transcriptional regulation, especially epigenetic regulation (Lisch and Bennetzen 2011). Transposons also can internally capture genes, can serve as sites of homology for unequal crossing-over, or can initiate a chromosome breakage-repair cycle (Wessler *et al.* 1995; Michelmore and Meyers 1998; Bennetzen 2005). All of these processes can amplify R-genes and can also move them to new genomic locations.

### R-gene nucleotide and haplotype diversity in switchgrass populations

Seven representative switchgrass populations were sampled from the upland and lowland ecotypes and the intermediate type, representing a broad geographic distribution. We detected various diversity patterns in different RGHs and different domains (NBS and LRR) within a resistance gene. The SwPc-1 RGH harbored low nucleotide diversity—only 0.51% on average at the population level. SwMLA-1 and SwRIII RGHs maintained an intermediate level of nucleotide diversity (3.5% and 2.74%), whereas SwPI-1 RGH demonstrated a greater level of diversity (7.2%). Many factors can affect genetic diversity, with some like population size relevant for all genes, whereas other factors like pathogen populations that are specific to each R-gene.

Nucleotide diversity also varied between populations. The upland SD population harbored the lowest diversity at the SwPc and SwMLA locus. This finding suggests that a less diverse set of pathogens recognized by these genes has been present in the upland environment, whereas the lowland environment might have had less diverse populations of the pathogens recognized by the SwPI-1 gene, which shows its lowest diversity in plants from these locations (Barrett *et al.* 2009; Ravensdale *et al.* 2011, 2012). Hence, this type of analysis might be useful to indicate locations and environments where it would be best to search both for the pathogens that these RGHs act against, and for the greatest sources of genetic diversity in resistance to these pathogens.

Overall, the average nucleotide diversity was greater in the lowland ecotypes than in the upland ecotypes for three of four RGHs. This is a different result from previous restriction fragment length polymorphism analysis of switchgrass ecotypes, where the upland ecotypes showed greater nucleotide diversity for the chloroplast *trnL* gene than the lowland ecotypes (64% *vs.* 56%) (Missaoui *et al.* 2006). The *trnL* gene has a conserved secondary structure and contains elements that are homologous across land plants (Hao *et al.* 2009). This observation suggests that lowland environments might contain a generally greater diversity of switchgrass pathogens.

Greater diversity was observed in the LRR domain in comparison to the NBS domain. This was the case for each of the RGHs, with the exception of SwRIII-1 where the LRR region targeted in this study was too short for adequate comparison. This difference is likely due to the different roles of the two domains, where the LRR domain is a recognition site adapting to new pathogen races and the NBS domain provides a conserved function in signal transduction. As shown in numerous other studies (Jiang *et al.* 2007; Sela *et al.* 2009), it is likely that the LRR region is more diverse than the NBS region in switchgrass RGHs because this is the region that interacts (directly or indirectly) with the corresponding *Avr* protein in the pathogen population (Hammond-Kosack and Jones 1997).

### Population structure of RGHs

AMOVA revealed that the majority of variance in haplotypic diversity was attributable to the within-population component, regardless of which populations were considered (Table S4). This is consistent with results from analysis of a full set of conserved grass EST-simple sequence repeat (SSR) markers from 31 switchgrass populations collected from 20 U.S. states (Narasimhamoorthy *et al.* 2008). This high within-population diversity may partly reflect balancing selection to increase within-population diversity as a mechanism of adaptive plasticity for disease resistance (Clay and Kover 1996) and responses to other environmental variables. As a near-obligate outcrosser, switchgrass is expected to have a very high level of gene flow, another mechanism for increasing diversity and for decreasing between-population variability. In this regard, switchgrass has a moderate *F*_st_ (0.19), similar to the mean value that has been observed for DNA markers (0.22) in 72 studies that analyzed the within population diversity for wild angiosperms and gymnosperms (Nybom 2004). Lower *F*_st_ values for RGHs than for molecular markers have also been reported in outcrossing pines (Diaz and Ferrer 2003) and outcrossing wild wheat (Sela *et al.* 2009), suggesting a particularly high level of selection for diversity *per se* in this category of genes.

In the current study, there was no evidence uncovered of an effect of isolation by distance on the population structure because the genetic distances did not correlate with geographic distances (*P* = 0.094). Detection of the selection factors affecting population structure is a difficult task when working with R-genes because many pathogens are involved, each responding differentially to environmental conditions. Although a significant correlation was not observed between geographic pattern and RGH diversity, the Northern-upland population from South Dakota (SD) demonstrated the lowest diversity for the SwPc and SwMLA RGHs whereas the largest genetic distance was observed with both upland and lowland Florida (FL) populations. Furthermore, from the PCoA analysis, it was observed that RGHs in the SD population did not cluster with RGHs from the other populations, suggesting a particularly high level of isolation for this population. Generally, upland ecotypes are adapted to the mid and Northern latitudes of the United States, whereas lowland ecotypes are adapted to the Southern U.S. RGHs identified in the Southern-upland (FL2) population originating from Florida were more closely related to another Southern-upland population from New Mexico (NM) rather than to a lowland population (FL1) from a nearby location. These results indicate, first, that adaptation to a specific growth environment (by, for instance, control of flowering time), rather than distance, plays a greater role in the RGH diversity distribution in switchgrass and, second, that gene flow between upland and lowland ecotypes may be quite restricted even in near-sympatric conditions.

### Evidence of balancing/positive selection and recombination

Thirteen sites in the SwPI and SwMLA RGHs were determined to be under positive selection. Among them, seven sites were in the LRR domain and six were in domain regions without a Pfam identification but adjacent to LRR domains. These results suggest that regions other than the LRR domain may also play a role in determining resistance specificity. There were no sites detected as under positive selection for the SwRIII and SwPc RGHs. This is likely to be a statistical issue deriving from the low nucleotide diversity at the SwPc locus and the relatively short LRR domain (39 bp) of the SwRIII-1 locus. Previous work suggests that selection pressure acts differently on different LRR modules. For example, 12 resistance gene loci from six plants revealed a significantly higher than neutral Ka/Ks value at the C-terminal region, whereas the N terminal region of the LRR flanking the β-strand/β-turn motif (××L×L××) exhibited purifying selection. In contrast, no significant Ka/Ks value (>1) was found between the N-terminal flanking and the non-LRR region or NBS domain that was predicted to be subject to purifying selection (Jiang *et al.* 2007). Numerous studies have shown that R-genes are subject to positive selection, especially in the LRR domain, where diversifying selection plays a role in the generation of new resistance specificities (Parniske *et al.* 1997; Ellis *et al.* 1999). There are also well-characterized examples of regions other than the LRR that contribute to resistance specificity. Studies of the *L* locus in flax indicated that the Toll interleukin-1 receptor domain contributes to resistance specificity and may be under positive selection (Luck *et al.* 2000), although no grass R-gene has yet been found with a Toll interleukin-1 receptor domain. A genome-wide study in Arabidopsis also indicated that approximately 30% of positively selected sites reside outside the LRRs, either in the NBS or in the loop region between the NBS and LRR (Mondragón-Palomino *et al.* 2002). This loop region between the NBS and LRR was the site of all four sites that we found to be under significant positive selection in the SwMLA locus.

We detected variability in the frequency of recombination events in the RGHs, indicating very different histories of sequence exchange between these loci. In recent decades, accumulating evidence suggests that unequal recombination is a major mechanism in diversifying RGH sequences (Parniske *et al.* 1997; Dixon *et al.* 1998; Hulbert *et al.* 2001; Nagy and Bennetzen 2008; Baurens *et al.* 2010). In maize, recombination has been shown to play an important role in the creation of genetic diversity at the *Rp1* rust resistance complex. *Rp1* haplotypes derived from unequal crossing-over, including in the LRR domain, have been found to lose race-specific resistance (reviewed in (Hulbert *et al.* 2001). Moreover, genetic analyses discovered four recombinant haplotypes from *Rp1* that conferred novel *Rp1* race specificities (Smith and Hulbert 2005). Similarly, extensive studies in flax, lettuce and tomato have suggested that recombination plays a central role in the evolution of new specificities (Parniske *et al.* 1997; Ellis *et al.* 1999; Luck *et al.* 2000; Chin *et al.* 2001). The very different rates of intragenic RGH discovered in this study suggest a combination of differences in selection frequency for recombined alleles (perhaps due to very different levels of pathogen stress) and different intrinsic levels of genetic instability (Bennetzen *et al.* 1988).

## Supplementary Material

Supporting Information
