# Supplementary material for: Isolation and Diversity Analysis of Resistance Gene Homologues from Switchgrass
Source: G3 (Bethesda). 2013 Jun 1;3(6):1031–42. doi: 10.1534/g3.112.005447 (PMC3689800; doi:10.1534/g3.112.005447)
Supplement: Supporting Information [file supp_3_6_1031__index.html]

Isolation and Diversity Analysis of Resistance Gene Homologues from Switchgrass — Supporting Information 

# Isolation and Diversity Analysis of Resistance Gene Homologues from Switchgrass

## Supporting Information for Zhu, Bennetzen, and Smith, 2013

**Files in this Data Supplement:**

- Supporting Information - Figures S1-S2 and Tables S1-S7 (PDF, 364 KB)
- Figure S1 - Gene structures of 12 fosmids containing NBS RGHs in switchgrass (PDF, 101 KB)
- Figure S2 - Neighbor-joining tree of 4 RGHs for 7 representative switchgrass populations (PDF, 446 KB)
- Table S1 - Primers used in the study (PDF, 101 KB)
- Table S2 - Summary statistics for 12 switchgrass fosmids containing NBS RGHs (PDF, 110 KB)
- Table S3 - Nucleotide diversity of NBS and LRR domains in switchgrass RGHs (PDF, 136 KB)
- Table S4 - Analysis of molecular variance (AMOVA) for RGHs in the switchgrass populations (PDF, 110 KB)
- Table S5 - Analysis of molecular variance (AMOVA) for RGHs in switchgrass populations and ecotypes (PDF, 109 KB)
- Table S6 - Summary of LRTs (Likelihood Ratio Tests) for positive selection in switchgrass RGHs (PDF, 110 KB)
- Table S7 - Candidate loci under positive selection (PDF, 110 KB)
