# Supplementary material for: Isolation and Diversity Analysis of Resistance Gene Homologues from Switchgrass
Source: G3 (Bethesda). 2013 Jun 1;3(6):1031–42. doi: 10.1534/g3.112.005447 (PMC3689800; doi:10.1534/g3.112.005447)
Supplement: Supporting Information [file supp_g3.112.005447_005447SI.pdf]

G3·Genes | Genomes | Genetics

Isolation and diversity analysis of resistance gene homologues from switchgrass

Qihui Zhu[1], Jeffrey L. Bennetzen[1] and Shavannor M. Smith[2]

[1]Department of Genetics, The University of Georgia, Athens, Georgia 30602, [2]Department of Plant Pathology, The University of Georgia, Athens, Georgia 30602

DOI: 10.1534/g3.112.005447

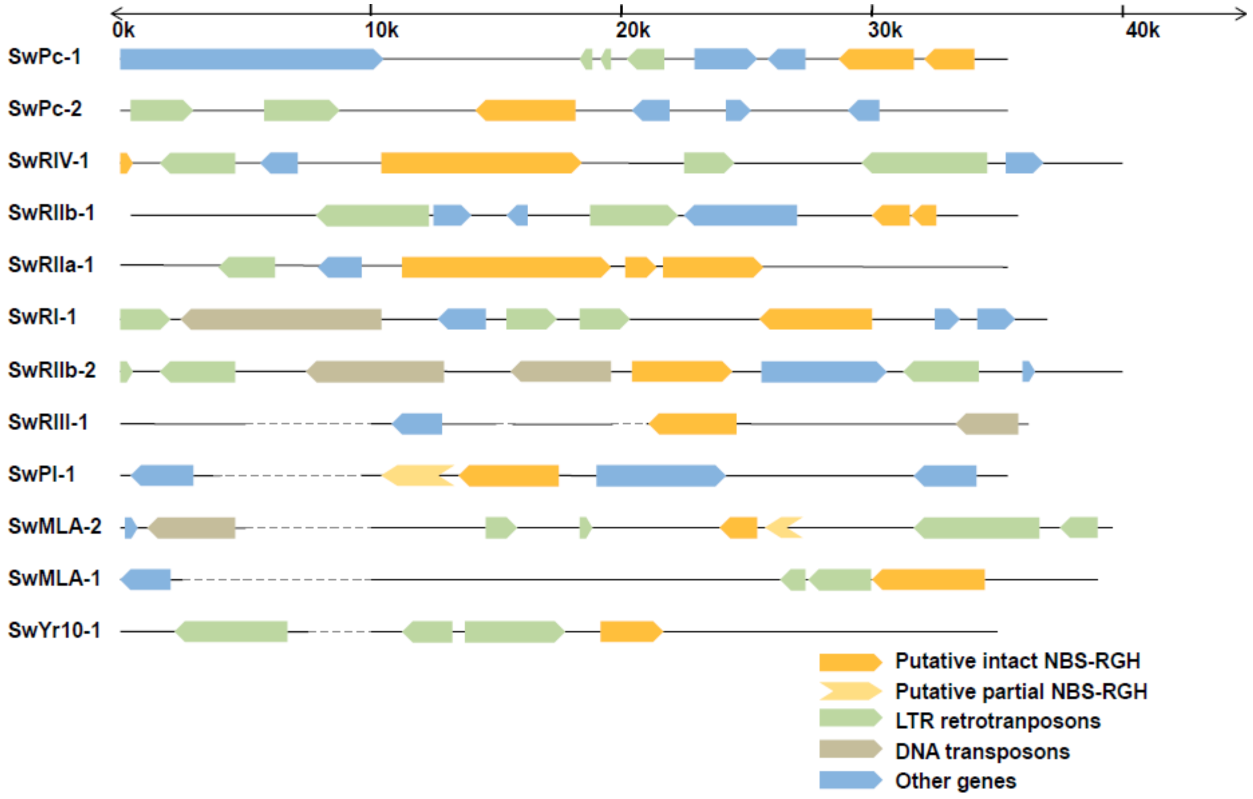

Figure S1 Gene structures of 12 fosmids containing NBS RGHs in switchgrass. Genes were predicted by gene finding programs FgeneSH and GeneMark.hmm. The unassembled contigs in SwRII-1, SwPI-1, SwMLA-1, SwMLA-2 and SwYr10-1 were represented with the grey dashed lines. Different colors represent different categories of genes (orange: putative intact NBS-RGHs, light orange: putative partial NBS-RGHs, light green: LTR retrotransposons, tan: DNA transposons, blue: all other genes). Number scale corresponds to the size of the fosmids in kilobases.

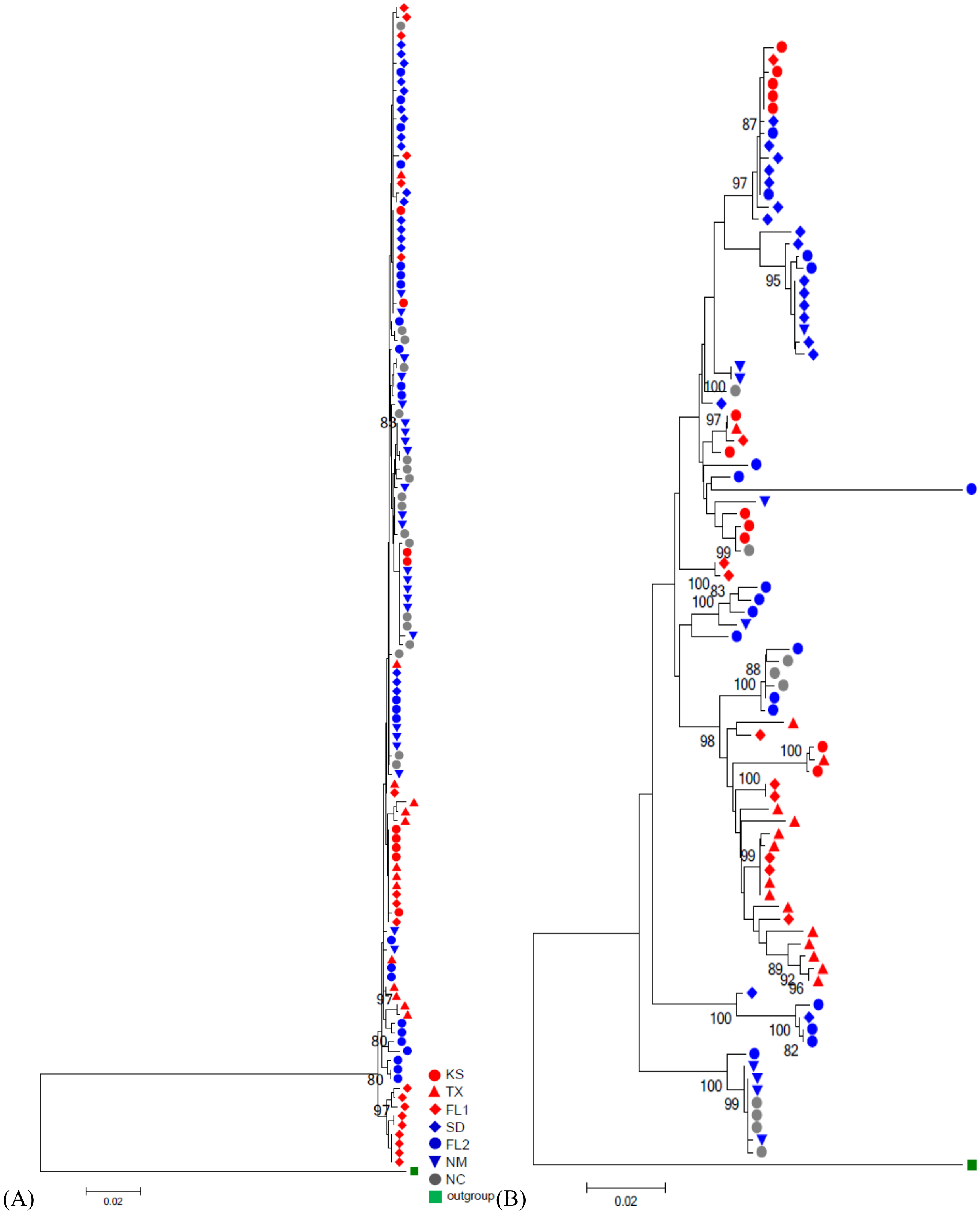
(A)
(B)
KS
TX
FL1
SD
FL2
NM
NC
outgroup
0.02
0.02

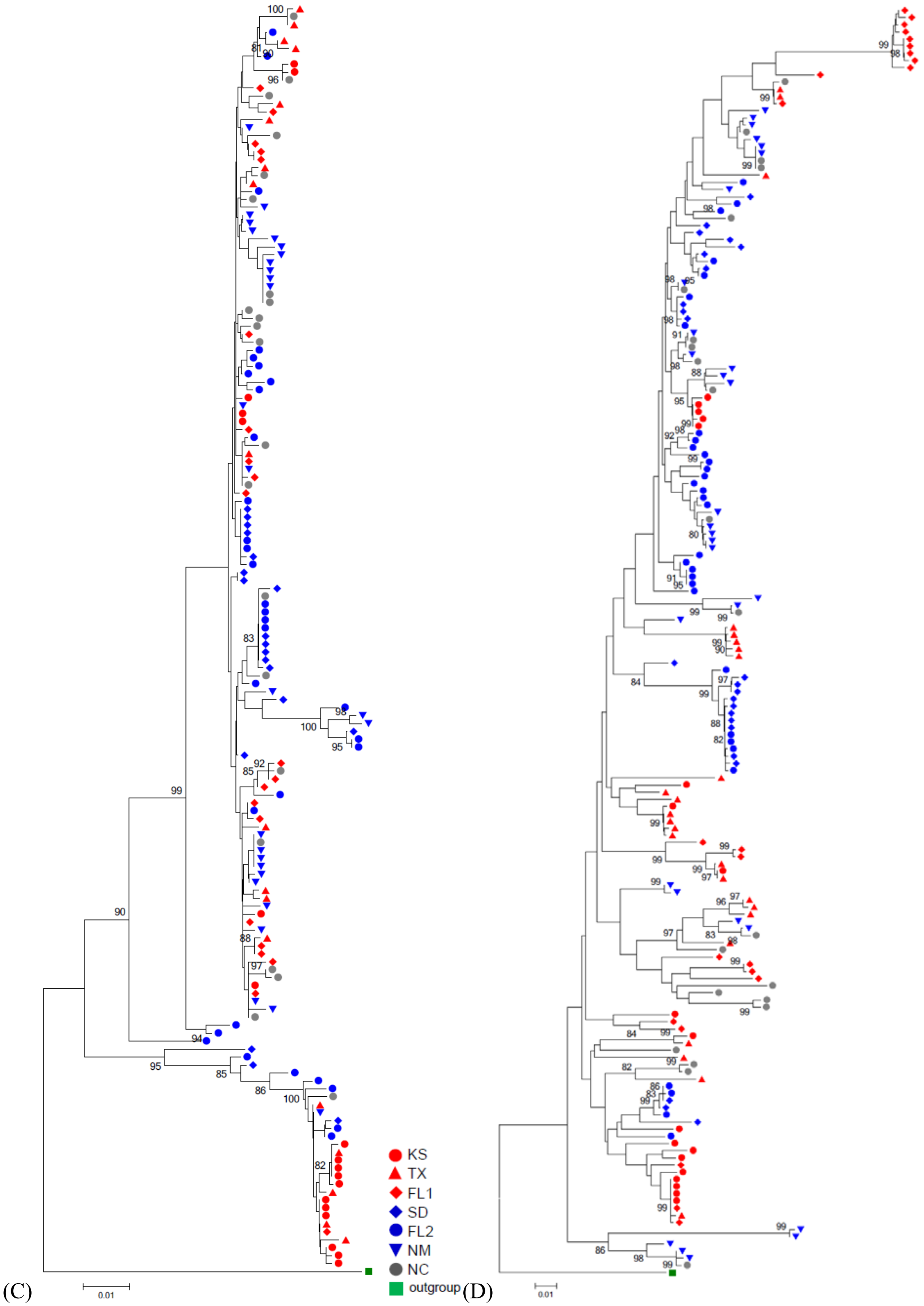

(C) (D)

Figure S2 Neighbor-joining tree of 4 RGHs for 7 representative switchgrass populations. (A) SwPc phylogenetical tree. (B) SwMLA phylogenetical tree. (C) SwRIII phylogenetical tree. (D) SwPI phylogenetical tree. Seven different signs represent 7 populations sampled from various geographic locations (KS- Kansas; TX- Texas; FL1-Florida; SD- South Dakota; FL2- Florida; NM-New Mexico; NC- North Carolina) for each locus. *Setaria italica* RGHs were used as the outgroup. Numbers at nodes indicate the level of branch support (%) with one-thousand bootstrap replicates.

Table S1 Primers used in the study

Name	Sequences (5'-3')	Orientation	Specificity
(Degenerate primers used to amplify putative NBS RGHs from genomic DNA)			
PloopF	ggHWtgggHggRWtRggVaag	Forward	PloopF
Kinase	ctBStYgtYYTsgATgAygT	Forward	Kinase/GLPLAL
GLPLAL	CARRgYCARWggAAgTCC	Reverse	PloopF/GLPLAL
MHD	ATCWYKWAgDWKRTCRTgCAT	Reverse	PloopF/MHD
Ploop1	ggIggI RTIggIAAIACIAC	Forward	Ploop1
GLP1	IAgIgYIAgIggIAgICC	Reverse	Ploop1/GLP1
GLP2	IATIgCIAgIggIAAICC	Reverse	Ploop1/GLP2
GLP3	IATIgCIAAIggIAgICC	Reverse	Ploop1/GLP3
GLP4	IAAIgYIAgIggIAgICC	Reverse	Ploop1/GLP4
GLP5	IAgIgCIAAIggIAgICC	Reverse	Ploop1/GLP5
GLP6	ARIgCTARIggIARICC	Reverse	Ploop1/GLP6
(Specific primers used to amplify target NBS RGHs from fosmid library)			
SwRI_F	GGGGTGGGGAAGACGACGCTAG	Forward	SwRI
SwRI_R	GGCGAGGGGGAAGCCTTTACAC	Reverse	SwRI
SwRIIa_F	GGGGTGGGGAAGACGACT	Forward	SwRIIa
SwRIIa_R	GAAGGGGAGGCCACCACAC	Reverse	SwRIIa
SwRIIb_F	GGGGGGTGGGGAAGACGACA	Forward	SwRIIb
SwRIIb_R	GYGAGGGGGAGGCCACCACATT	Reverse	SwRIIb
SwRIII_F	GGGTGGGGAAGACGACGTT	Forward	SwRIII
SwRIII_R	GGGGAGGCCTCCACATTTCT	Reverse	SwRIII
SwRIV_F	GGGGTGGGGAAGACGACAC	Forward	SwRIV
SwRIV_R	GAGGGGGAGGCSTGCACACT	Reverse	SwRIV
SwPc_F	GGTGGGTCTGGMAAAACTAC	Forward	SwPc
SwPc_R	TGATTGCTAGwGGCACCC	Reverse	SwPc
SwPI_F	GGACTTCCCCTTGCAATMGT	Forward	SwPI
SwPI_R	GATCRTGGASYTTGCAAGAG	Reverse	SwPI
SwMLA_F	GTGGYTGYCTTCCTCTTGC	Forward	SwMLA
SwMLA_R	GYTGYCTYASWAGGTCATGC	Reverse	SwMLA
SwYr_F	GGAGGACTMGGCAAGACAAC	Forward	SwYr
SwYr_R	CGATAGCTAACGGGACACCT	Reverse	SwYr
(Specific primers used to amplify target NBS RGHs from switchgrass populations)			
SwPc_PF	CCCGAAGGTTTGGGCATTGA	Forward	SwPc
SwPc_PR	CCGGCAACACCGTGAGATTA	Reverse	SwPc
SwPI_PF	CGGAGGCGTTTGGTGCATCG	Forward	SwPI
SwPI_PR	ACTTGCGCAGTCCCGTGAGC	Reverse	SwPI
SwMLA_PF	TGGACGTAGGATGGGAGCTGCTT	Forward	SwMLA
SwMLA_PR	TGCAAAGAGTGGCATCCCTGTAAG	Reverse	SwMLA
SwRIII_PF	TCCGAGGACTGCTGCCCTGAT	Forward	SwRIII
SwRIII_PR	ACATGTCAGTGCCCCTGAGGCT	Reverse	SwRIII

Table S2 Summary statistics for 12 switchgrass fosmids containing NBS RGHs

Fosmid Name	Sequenced fosmid code	Unassembled contig number	No. of Genes [a]	No. of intact NBS-RGHs	No. of partial NBS-RGHs [b]	TE Number [c]
SwRI-1	4086665	1	8	1	0	4
SwRIIa-1	4086670	1	5	3	0	1
SwRIIb-1	4087545	1	7	2	0	2
SwRIIb-2	4087546	1	8	1	0	5
SwRIII-1	4086668	5	3	1	0	1
SwRIV-1	4086669	1	7	2	0	3
SwPc-1	4086666	1	8	2	0	3
SwPc-2	4086667	1	6	1	0	2
SwPI-1	4087536	3	4	1	1	0
SwMLA-1	4087537	2	4	1	0	2
SwMLA-2	4087538	2	8	1	1	5
SwYr10-1	4087539	4	4	1	0	3

[a] Genes on each fosmid were predicted by Fgenesh and GeneMark. The number of genes includes RGHs and other genes, but not TE-related genes.
[b] No. of partial NBS-RGHs were predicted by HMMER search.
[c] Transposable elements (TEs) included in the fosmid. No genes were identified inside the predicted TEs, presumably due to their truncated and/or pseudogene status.

Table S3 Nucleotide diversity of NBS and LRR domains in switchgrass RGHs

Gene	GenBank [a]	Domain [b]	Region [c]	Sites [d]	S [e]	h [f]	H_d [g]	π [h]	θ_w [i]
SwPc	JN231832 - JN231957	NBS	1-342	342	65	23	0.818	0.83%	3.85%
		LRR	740-1024	285	77	21	0.815	0.90%	5.37%
		Total	1-1024	1024	250	60	0.961	0.88%	5.01%
SwRIII	JN231684 - JN231831	NBS	1-351	351	77	61	0.955	3.28%	4.44%
		LRR	776-814	39	6	5	0.313	0.95%	2.72%
		Total	1-814	814	195	112	0.99	3.32%	4.98%
SwMLA	JN231958 - JN232038	NBS	1-387	387	131	52	0.971	4.38%	7.87%
		LRR	723-896	174	64	32	0.905	2.69%	8.11%
		Total	1-896	896	329	68	0.988	4.52%	8.83%
SwPI	JN231541 - JN231683	NBS	1-90	90	41	36	0.912	4.60%	9.65%
		LRR	494-1024	531	199	103	0.988	10.10%	13.68%
		Total	1-1024	1024	431	145	0.996	7.80%	12.19%

[a] GenBank accession numbers for this RGH

[b] NBS represents nucleotide binding site and LRR represents leucine-rich repeat domain.

[c] Range of the domain included in the aligned dataset.

[d] Number of nucleotides in the analyzed domain.

[e] Number of segregating (polymorphic) sites measured with the Nei method (Nei, 1987).

[f] Number of unique haplotypes measured with the Nei method (Nei, 1987).

[g] Frequency of a haplotype in a sample, $H_d = (1 - \Sigma(x_i)^2)\ n / (n - 1)$, where x_i is the frequency of a haplotype and n is the sample size, measured with the Nei method (Nei, 1987).

[h] Average number of nucleotide differences per site between two sequences (nucleotide diversity) measured with the Nei method (Nei, 1987).

[i] Number of segregating sites in a sample predicted with Watterson's estimator (θ_w). $\theta = 4N_e u$ for an autosomal gene of a diploid organism where N_e and u are the effective population size and the mutation rate per DNA sequence per generation, respectively, measured with the Watterson method (Watterson 1975).

Table S4 Analysis of molecular variance (AMOVA) for RGHs in the switchgrass populations

Gene	Source	df [a]	SS [b]	MS [c]	Est. Var [d].	% total [e]
SwPc	Between Pops	6	96.644	16.107	0.713	20%
	Within Pops	124	359.050	2.896	2.896	80%
	Total	130	455.695	N/A	3.609	100%
SwRIII	Between Pops	6	373.306	62.218	2.140	16%
	Within Pops	160	1845.065	11.532	11.532	84%
	Total	166	2218.371	N/A	13.671	100%
SwMLA	Between Pops	6	355.885	59.314	3.017	16%
	Within Pops	94	1531.897	16.297	16.297	84%
	Total	100	1887.782	N/A	19.314	100%
SwPI	Between Pops	6	1117.589	186.265	6.410	14%
	Within Pops	157	6019.033	38.338	38.338	86%
	Total	163	7136.622	N/A	44.747	100%

All parameters were measured with the Analysis of molecular variance (AMOVA) method.
[a] Degrees of freedom.
[b] Sum of squares.
[c] Mean square deviations.
[d] Estimated variance of component.
[e] Percentage of total variation.

Table S5 Analysis of molecular variance (AMOVA) for RGHs in switchgrass populations and ecotypes

Gene	Source of Variation	*d.f.* [a]	x^2	Variation
SwPc	Between ecotypes	2	42.4	5%
	Between populations within ecotype	4	56.5	18%
	Within populations	119	334.3	77%
SwMLA	Between ecotypes	2	154.5	5%
	Between populations within ecotype	4	225.9	15%
	Within populations	88	1381.0	80%
SwRIII	Between ecotypes	2	105.3	0%
	Between populations within ecotype	4	253.9	17%
	Within populations	153	1754.3	83%
SwPI	Between ecotypes	2	539.5	6%
	Between populations within ecotype	4	578.1	10%
	Within populations	157	6019.1	84%

[a] degrees of freedom.

Table S6 Summary of LRTs (Likelihood Ratio Tests) for positive selection in switchgrass RGHs

Gene	Length [a]	M2 [b]	M1 [c]	2*L* [d]	*P* -value	Positive selection
SwPC	990	-2283.4	-2286.5	6.3	0.04	-
SwRIII	806	-2240.7	-2240.7	0.0	1.00	-
SwMLA	894	-3174.6	-3208.8	68.4	1.4E-15	YES
SwPI	1023	-6682.8	-6771.0	176.5	4.8E-39	YES

[a]Aligned length of sequences.
[b]Model designates selection in PAML (Yang 1997).
[c]Model designates neutral in PAML (Yang 1997).
[d] Likelihood ratio.

Table S7 Candidate loci under positive selection

Gene	AA Position [a]	AA	Posterior Probability [b]	Location [c]
SwMLA	147	M	1.000**	Loop
SwMLA	150	R	1.000**	Loop
SwMLA	151	L	1.000**	Loop
SwMLA	227	Q	0.953*	Loop
SwPI	123	K	0.997**	Loop
SwPI	143	T	0.979*	Loop
SwPI	190	W	1.000**	LRR
SwPI	192	N	1.000**	LRR
SwPI	210	R	1.000**	LRR
SwPI	216	T	1.000**	LRR
SwPI	231	V	1.000**	LRR
SwPI	232	E	1.000**	LRR
SwPI	254	R	0.990*	LRR

[a]Amino acid position in the aligned dataset.
[b]*P>95%; **P>99%.
[c]Location of amino acid under selection in the corresponding domain, LRR represents the leucine-rich repeat domain and Loop represents the region between the NBS and LRR domain.
