# Supplementary material for: Isolation and Diversity Analysis of Resistance Gene Homologues from Switchgrass
Source: G3 (Bethesda). 2013 Jun 1;3(6):1031–42. doi: 10.1534/g3.112.005447 (PMC3689800; doi:10.1534/g3.112.005447)
Supplement: Supporting Information [file supp_g3.112.005447_FigureS1.pdf]

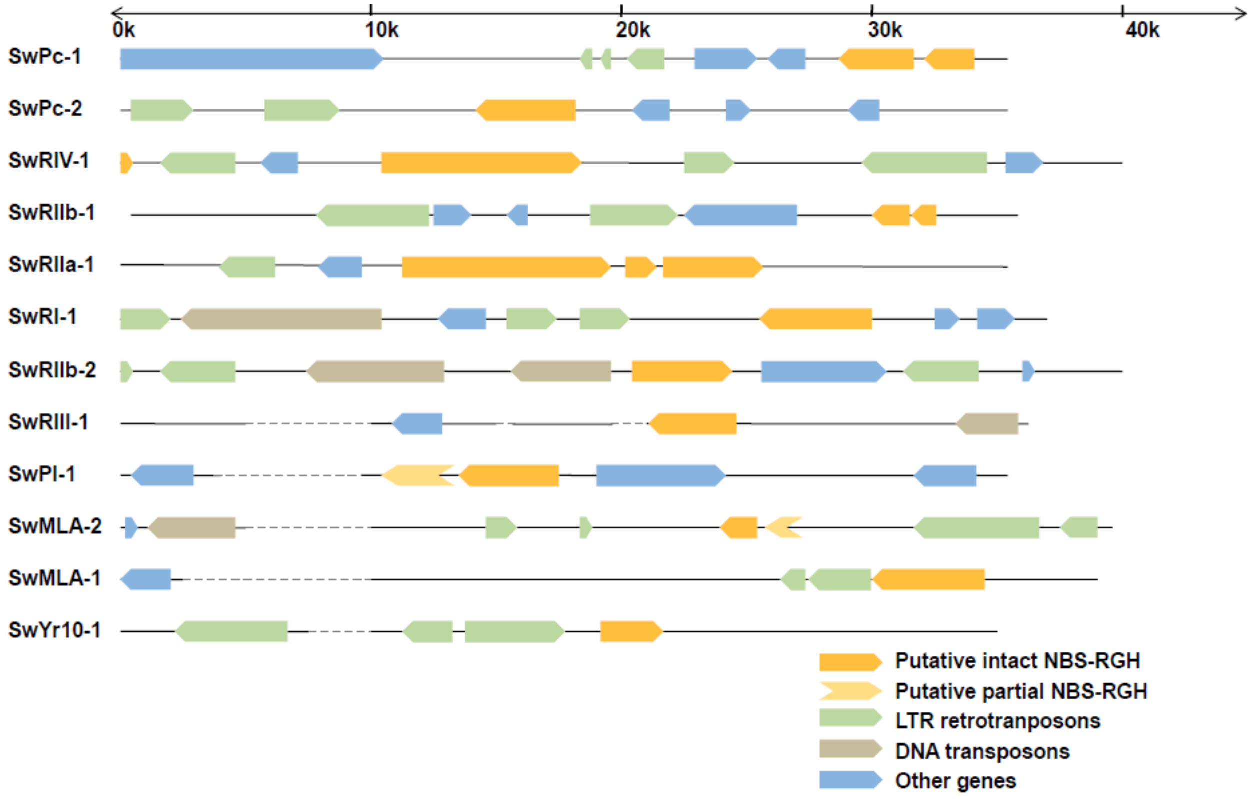

Figure S1 Gene structures of 12 fosmids containing NBS RGHs in switchgrass. Genes were predicted by gene finding programs FgeneSH and GeneMark.hmm. The unassembled contigs in SwRII-1, SwPI-1, SwMLA-1, SwMLA-2 and SwYr10-1 were represented with the grey dashed lines. Different colors represent different categories of genes (orange: putative intact NBS-RGHs, light orange: putative partial NBS-RGHs, light green: LTR retrotransposons, tan: DNA transposons, blue: all other genes). Number scale corresponds to the size of the fosmids in kilobases.
