# Supplementary material for: Isolation and Diversity Analysis of Resistance Gene Homologues from Switchgrass
Source: G3 (Bethesda). 2013 Jun 1;3(6):1031–42. doi: 10.1534/g3.112.005447 (PMC3689800; doi:10.1534/g3.112.005447)
Supplement: Supporting Information [file supp_g3.112.005447_FigureS2.pdf]

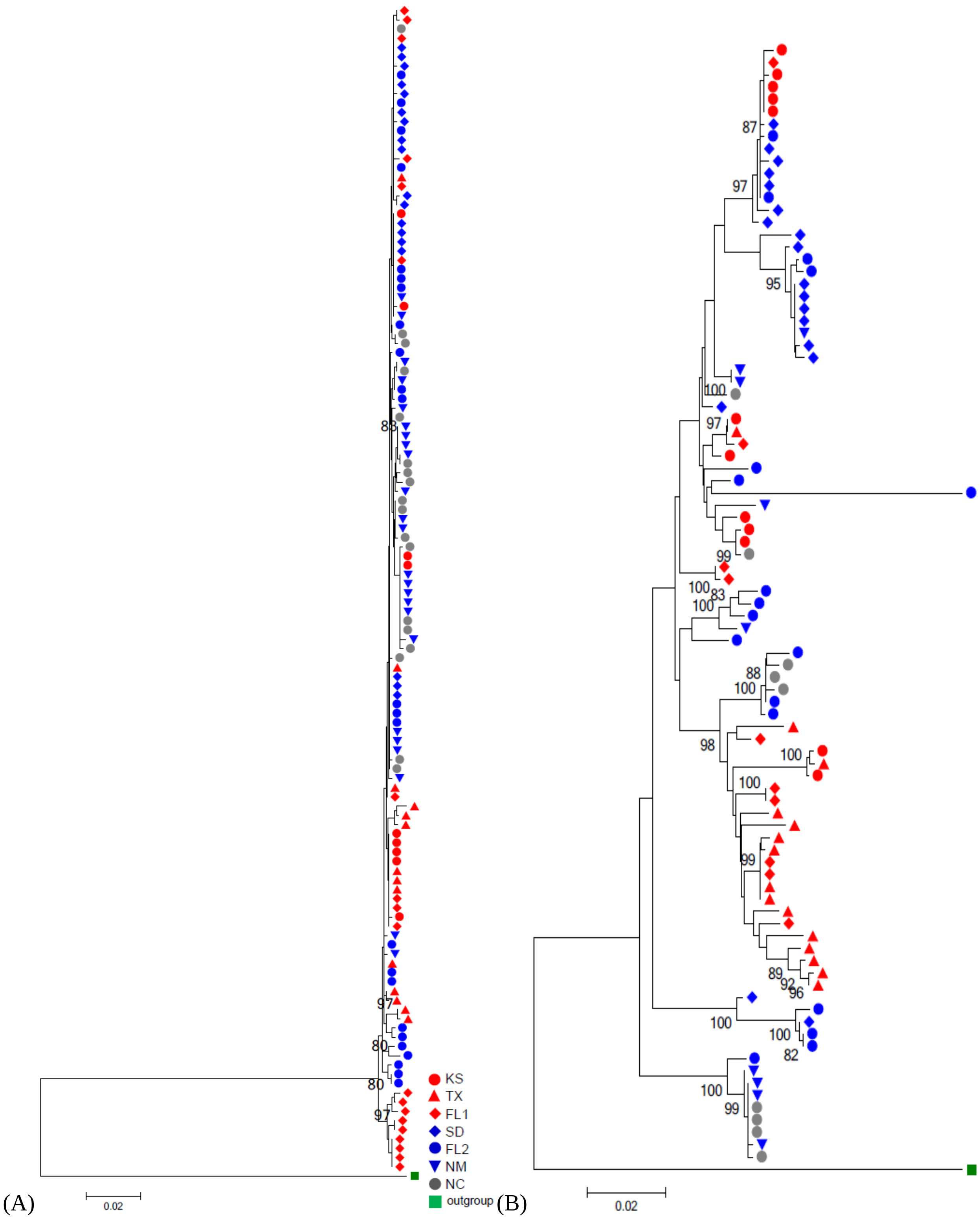

Q. Zhu, J. L. Bennetzen, and S. M. Smith

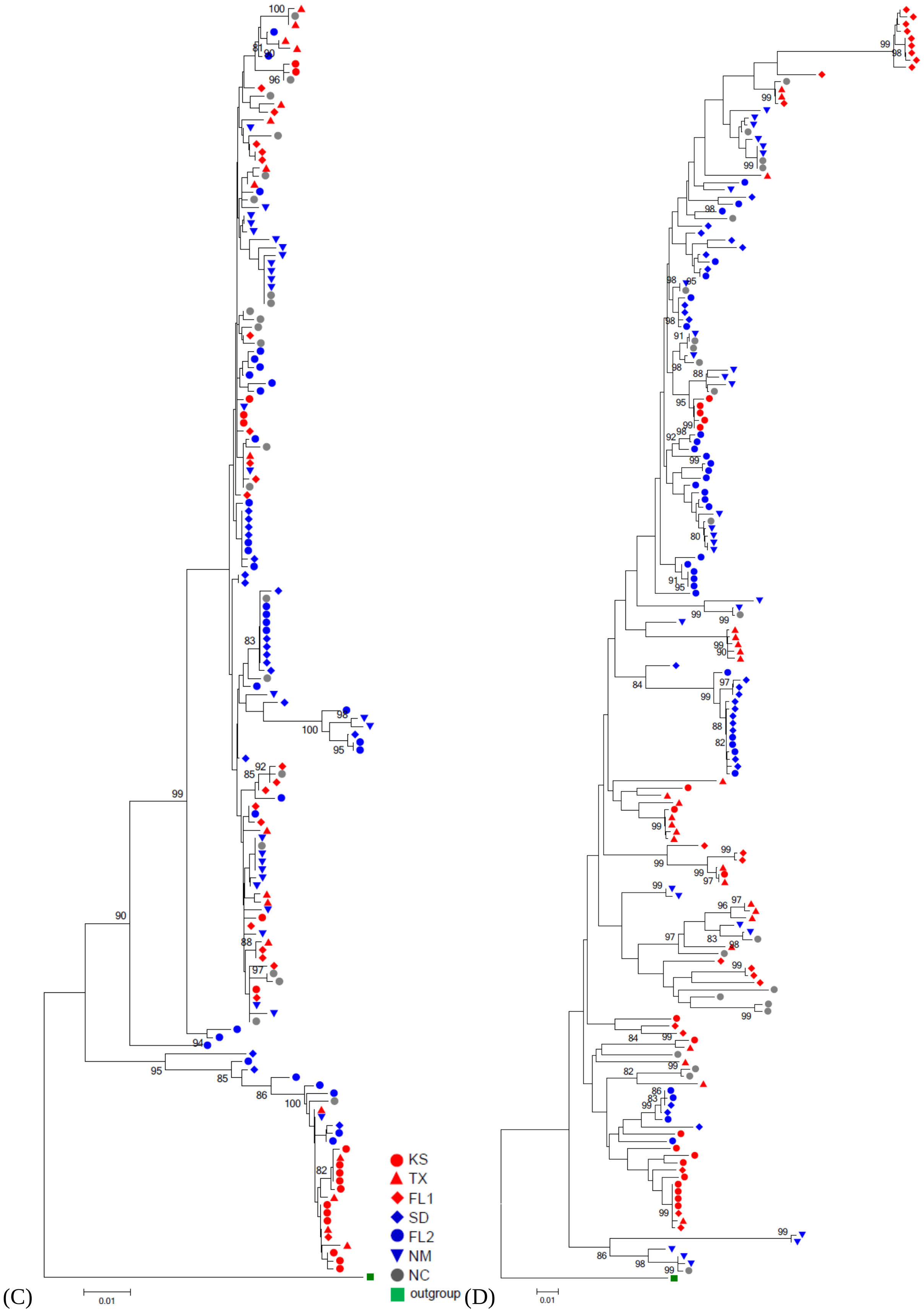
KS
TX
FL1
SD
FL2
NM
NC
outgroup
0.01
(C)
(D)

Figure S2 Neighbor-joining tree of 4 RGHs for 7 representative switchgrass populations. (A) SwPc phylogenetical tree. (B) SwMLA phylogenetical tree. (C) SwRIII phylogenetical tree. (D) SwPI phylogenetical tree. Seven different signs represent 7 populations sampled from various geographic locations (KS- Kansas; TX- Texas; FL1-Florida; SD- South Dakota; FL2- Florida; NM-New Mexico; NC- North Carolina) for each locus. *Setaria italica* RGHs were used as the outgroup. Numbers at nodes indicate the level of branch support (%) with one-thousand bootstrap replicates.
