# Supplementary material for: Isolation and Diversity Analysis of Resistance Gene Homologues from Switchgrass
Source: G3 (Bethesda). 2013 Jun 1;3(6):1031–42. doi: 10.1534/g3.112.005447 (PMC3689800; doi:10.1534/g3.112.005447)
Supplement: Supporting Information [file supp_g3.112.005447_TableS1.pdf]

Table S1 Primers used in the study

Name	Sequences (5'-3')	Orientation	Specificity
(Degenerate primers used to amplify putative NBS RGHs from genomic DNA)			
PloopF	ggHWtgggHggRWtRggVaag	Forward	PloopF
Kinase	ctBStYgtYYTsgATgAygT	Forward	Kinase/GLPLAL
GLPLAL	CARRgYCARWggAAgTCC	Reverse	PloopF/GLPLAL
MHD	ATCWYKWAgDWKRTCRTgCAT	Reverse	PloopF/MHD
Ploop1	ggIggI RTIggIAAIACIAC	Forward	Ploop1
GLP1	IAgIgYIAgIggIAgICC	Reverse	Ploop1/GLP1
GLP2	IATIgCIAgIggIAAICC	Reverse	Ploop1/GLP2
GLP3	IATIgCIAAIggIAgICC	Reverse	Ploop1/GLP3
GLP4	IAAIgYIAgIggIAgICC	Reverse	Ploop1/GLP4
GLP5	IAgIgCIAAIggIAgICC	Reverse	Ploop1/GLP5
GLP6	ARIgCTARIggIARICC	Reverse	Ploop1/GLP6
(Specific primers used to amplify target NBS RGHs from fosmid library)			
SwRI_F	GGGGTGGGGAAGACGACGCTAG	Forward	SwRI
SwRI_R	GGCGAGGGGGAAGCCTTTACAC	Reverse	SwRI
SwRIIa_F	GGGGTGGGGAAGACGACT	Forward	SwRIIa
SwRIIa_R	GAAGGGGAGGCCACCACAC	Reverse	SwRIIa
SwRIIb_F	GGGGGGTGGGGAAGACGACA	Forward	SwRIIb
SwRIIb_R	GYGAGGGGGAGGCCACCACATT	Reverse	SwRIIb
SwRIII_F	GGGTGGGGAAGACGACGTT	Forward	SwRIII
SwRIII_R	GGGGAGGCCTCCACATTTCT	Reverse	SwRIII
SwRIV_F	GGGGTGGGGAAGACGACAC	Forward	SwRIV
SwRIV_R	GAGGGGGAGGCSTGCACACT	Reverse	SwRIV
SwPc_F	GGTGGGTCTGGMAAAACTAC	Forward	SwPc
SwPc_R	TGATTGCTAGwGGCACCC	Reverse	SwPc
SwPI_F	GGACTTCCCCTTGCAATMGT	Forward	SwPI
SwPI_R	GATCRTGGASYTTGCAAGAG	Reverse	SwPI
SwMLA_F	GTGGYTGYCTTCCTCTTGC	Forward	SwMLA
SwMLA_R	GYTGYCTYASWAGGTCATGC	Reverse	SwMLA
SwYr_F	GGAGGACTMGGCAAGACAAC	Forward	SwYr
SwYr_R	CGATAGCTAACGGGACACCT	Reverse	SwYr
(Specific primers used to amplify target NBS RGHs from switchgrass populations)			
SwPc_PF	CCCGAAGGTTTGGGCATTGA	Forward	SwPc
SwPc_PR	CCGGCAACACCGTGAGATTA	Reverse	SwPc
SwPI_PF	CGGAGGCGTTTGGTGCATCG	Forward	SwPI
SwPI_PR	ACTTGCGCAGTCCCGTGAGC	Reverse	SwPI
SwMLA_PF	TGGACGTAGGATGGGAGCTGCTT	Forward	SwMLA
SwMLA_PR	TGCAAAGAGTGGCATCCCTGTAAG	Reverse	SwMLA
SwRIII_PF	TCCGAGGACTGCTGCCCTGAT	Forward	SwRIII
SwRIII_PR	ACATGTCAGTGCCCCTGAGGCT	Reverse	SwRIII
