# Supplementary material for: Isolation and Diversity Analysis of Resistance Gene Homologues from Switchgrass
Source: G3 (Bethesda). 2013 Jun 1;3(6):1031–42. doi: 10.1534/g3.112.005447 (PMC3689800; doi:10.1534/g3.112.005447)
Supplement: Supporting Information [file supp_g3.112.005447_TableS2.pdf]

Table S2 Summary statistics for 12 switchgrass fosmids containing NBS RGHs

Fosmid Name	Sequenced fosmid code	Unassembled contig number	No. of Genes [a]	No. of intact NBS-RGHs	No. of partial NBS-RGHs [b]	TE Number [c]
SwRI-1	4086665	1	8	1	0	4
SwRIIa-1	4086670	1	5	3	0	1
SwRIIb-1	4087545	1	7	2	0	2
SwRIIb-2	4087546	1	8	1	0	5
SwRIII-1	4086668	5	3	1	0	1
SwRIV-1	4086669	1	7	2	0	3
SwPc-1	4086666	1	8	2	0	3
SwPc-2	4086667	1	6	1	0	2
SwPI-1	4087536	3	4	1	1	0
SwMLA-1	4087537	2	4	1	0	2
SwMLA-2	4087538	2	8	1	1	5
SwYr10-1	4087539	4	4	1	0	3

[a] Genes on each fosmid were predicted by Fgenesh and GeneMark. The number of genes includes RGHs and other genes, but not TE-related genes.
[b] No. of partial NBS-RGHs were predicted by HMMER search.
[c] Transposable elements (TEs) included in the fosmid. No genes were identified inside the predicted TEs, presumably due to their truncated and/or pseudogene status.
