# Supplementary material for: Isolation and Diversity Analysis of Resistance Gene Homologues from Switchgrass
Source: G3 (Bethesda). 2013 Jun 1;3(6):1031–42. doi: 10.1534/g3.112.005447 (PMC3689800; doi:10.1534/g3.112.005447)
Supplement: Supporting Information [file supp_g3.112.005447_TableS3.pdf]

Table S3 Nucleotide diversity of NBS and LRR domains in switchgrass RGHs

Gene	GenBank [a]	Domain [b]	Region [c]	Sites [d]	S [e]	h [f]	H_d [g]	π [h]	θ_w [i]
SwPc	JN231832 - JN231957	NBS	1-342	342	65	23	0.818	0.83%	3.85%
		LRR	740-1024	285	77	21	0.815	0.90%	5.37%
		Total	1-1024	1024	250	60	0.961	0.88%	5.01%
SwRIII	JN231684 - JN231831	NBS	1-351	351	77	61	0.955	3.28%	4.44%
		LRR	776-814	39	6	5	0.313	0.95%	2.72%
		Total	1-814	814	195	112	0.99	3.32%	4.98%
SwMLA	JN231958 - JN232038	NBS	1-387	387	131	52	0.971	4.38%	7.87%
		LRR	723-896	174	64	32	0.905	2.69%	8.11%
		Total	1-896	896	329	68	0.988	4.52%	8.83%
SwPI	JN231541 - JN231683	NBS	1-90	90	41	36	0.912	4.60%	9.65%
		LRR	494-1024	531	199	103	0.988	10.10%	13.68%
		Total	1-1024	1024	431	145	0.996	7.80%	12.19%

[a] GenBank accession numbers for this RGH

[b] NBS represents nucleotide binding site and LRR represents leucine-rich repeat domain.

[c] Range of the domain included in the aligned dataset.

[d] Number of nucleotides in the analyzed domain.

[e] Number of segregating (polymorphic) sites measured with the Nei method (Nei, 1987).

[f] Number of unique haplotypes measured with the Nei method (Nei, 1987).

[g] Frequency of a haplotype in a sample, $H_d = (1 - \Sigma(x_i)^2)\, n / (n - 1)$, where x_i is the frequency of a haplotype and n is the sample size, measured with the Nei method (Nei, 1987).

[h] Average number of nucleotide differences per site between two sequences (nucleotide diversity) measured with the Nei method (Nei, 1987).

[i] Number of segregating sites in a sample predicted with Watterson's estimator (θ_w). $\theta = 4N_e u$ for an autosomal gene of a diploid organism where N_e and u are the effective population size and the mutation rate per DNA sequence per generation, respectively, measured with the Watterson method (Watterson 1975).
