# Supplementary material for: Isolation and Diversity Analysis of Resistance Gene Homologues from Switchgrass
Source: G3 (Bethesda). 2013 Jun 1;3(6):1031–42. doi: 10.1534/g3.112.005447 (PMC3689800; doi:10.1534/g3.112.005447)
Supplement: Supporting Information [file supp_g3.112.005447_TableS4.pdf]

Table S4 Analysis of molecular variance (AMOVA) for RGHs in the switchgrass populations

Gene	Source	df [a]	SS [b]	MS [c]	Est. Var [d].	% total [e]
SwPc	Between Pops	6	96.644	16.107	0.713	20%
	Within Pops	124	359.050	2.896	2.896	80%
	Total	130	455.695	N/A	3.609	100%
SwRIII	Between Pops	6	373.306	62.218	2.140	16%
	Within Pops	160	1845.065	11.532	11.532	84%
	Total	166	2218.371	N/A	13.671	100%
SwMLA	Between Pops	6	355.885	59.314	3.017	16%
	Within Pops	94	1531.897	16.297	16.297	84%
	Total	100	1887.782	N/A	19.314	100%
SwPI	Between Pops	6	1117.589	186.265	6.410	14%
	Within Pops	157	6019.033	38.338	38.338	86%
	Total	163	7136.622	N/A	44.747	100%

All parameters were measured with the Analysis of molecular variance (AMOVA) method.
[a] Degrees of freedom.
[b] Sum of squares.
[c] Mean square deviations.
[d] Estimated variance of component.
[e] Percentage of total variation.
