# Supplementary material for: Isolation and Diversity Analysis of Resistance Gene Homologues from Switchgrass
Source: G3 (Bethesda). 2013 Jun 1;3(6):1031–42. doi: 10.1534/g3.112.005447 (PMC3689800; doi:10.1534/g3.112.005447)
Supplement: Supporting Information [file supp_g3.112.005447_TableS5.pdf]

Table S5 Analysis of molecular variance (AMOVA) for RGHs in switchgrass populations and ecotypes

Gene	Source of Variation	*d.f.* [a]	x^2	Variation
SwPc	Between ecotypes	2	42.4	5%
	Between populations within ecotype	4	56.5	18%
	Within populations	119	334.3	77%
SwMLA	Between ecotypes	2	154.5	5%
	Between populations within ecotype	4	225.9	15%
	Within populations	88	1381.0	80%
SwRIII	Between ecotypes	2	105.3	0%
	Between populations within ecotype	4	253.9	17%
	Within populations	153	1754.3	83%
SwPI	Between ecotypes	2	539.5	6%
	Between populations within ecotype	4	578.1	10%
	Within populations	157	6019.1	84%

[a] degrees of freedom.
