# Supplementary material for: Isolation and Diversity Analysis of Resistance Gene Homologues from Switchgrass
Source: G3 (Bethesda). 2013 Jun 1;3(6):1031–42. doi: 10.1534/g3.112.005447 (PMC3689800; doi:10.1534/g3.112.005447)
Supplement: Supporting Information [file supp_g3.112.005447_TableS6.pdf]

Table S6 Summary of LRTs (Likelihood Ratio Tests) for positive selection in switchgrass RGHs

Gene	Length [a]	M2 [b]	M1 [c]	2*L* [d]	*P* -value	Positive selection
SwPC	990	-2283.4	-2286.5	6.3	0.04	-
SwRIII	806	-2240.7	-2240.7	0.0	1.00	-
SwMLA	894	-3174.6	-3208.8	68.4	1.4E-15	YES
SwPI	1023	-6682.8	-6771.0	176.5	4.8E-39	YES

[a]Aligned length of sequences.
[b]Model designates selection in PAML (Yang 1997).
[c]Model designates neutral in PAML (Yang 1997).
[d] Likelihood ratio.
