# Supplementary material for: Isolation and Diversity Analysis of Resistance Gene Homologues from Switchgrass
Source: G3 (Bethesda). 2013 Jun 1;3(6):1031–42. doi: 10.1534/g3.112.005447 (PMC3689800; doi:10.1534/g3.112.005447)
Supplement: Supporting Information [file supp_g3.112.005447_TableS7.pdf]

Table S7 Candidate loci under positive selection

Gene	AA Position [a]	AA	Posterior Probability [b]	Location [c]
SwMLA	147	M	1.000**	Loop
SwMLA	150	R	1.000**	Loop
SwMLA	151	L	1.000**	Loop
SwMLA	227	Q	0.953*	Loop
SwPI	123	K	0.997**	Loop
SwPI	143	T	0.979*	Loop
SwPI	190	W	1.000**	LRR
SwPI	192	N	1.000**	LRR
SwPI	210	R	1.000**	LRR
SwPI	216	T	1.000**	LRR
SwPI	231	V	1.000**	LRR
SwPI	232	E	1.000**	LRR
SwPI	254	R	0.990*	LRR

[a]Amino acid position in the aligned dataset.

[b]*P>95%; **P>99%.

[c]Location of amino acid under selection in the corresponding domain, LRR represents the leucine-rich repeat domain and Loop represents the region between the NBS and LRR domain.
